# Exploring *Bacillus thuringiensis* as a model for endospore adhesion and its potential to investigate adhesins in *Pasteuria penetrans*

**DOI:** 10.1111/jam.15522

**Published:** 2022-03-22

**Authors:** Arohi Srivastava, Sharad Mohan, Keith G. Davies

**Affiliations:** ^1^ School of Life and Medical Sciences University of Hertfordshire Hatfield UK; ^2^ Division of Nematology Indian Agricultural Research Institute New Delhi India

**Keywords:** biological control, collagen‐like proteins, crop protection, glycosylation, host specificity, immunofluorescence, *Meloidogyne*, Western blot analysis

## Abstract

**Aims:**

Phytonematodes are a constraint on crop production and have been controlled using nematicides; these are highly toxic and legislation in Europe and elsewhere is prohibiting their use and alternatives are being sought. *Pasteuria penetrans* is a hyperparasitic bacterium that form endospores and have potential to control root‐knot nematodes (*Meloidogyne* spp.), but their attachment to the nematode cuticle is host‐specific. Understanding host specificity has relied upon endospore inhibition bioassays using immunological and biochemical approaches. Phylogenetic analysis of survey sequences has shown *P. penetrans* to be closely related to *Bacillus* and to have a diverse range of collagen‐like fibres which we hypothesise to be involved in the endospore adhesion. However, due to the obligately hyperparasitic nature of *Pasteuria* species, identifying and characterizing these collagenous‐like proteins through gain of function has proved difficult and new approaches are required.

**Methods and Results:**

Using antibodies raised to synthetic peptides based on *Pasteuria* collagen‐like genes we show similarities between *P. penetrans* and the more easily cultured bacterium *Bacillus thuringiensis* and suggest it be used as a gain of function platform/model. Using immunological approaches similar proteins between *P. penetrans* and *B. thuringiensis* are identified and characterized, one >250 kDa and another ~72 kDa are glycosylated with N‐acetylglucosamine and both of which are digested if treated with collagenase. These treatments also affected endospore attachment and suggest these proteins are involved in adhesion of endospores to nematode cuticle.

**Conclusion:**

There are conserved similarities in the collagen‐like proteins present on the surface of endospores of both *P. penetrans* and *B. thuringiensis*.

**Significance and Impact of Study:**

As *B. thuringiensis* is relatively easy to culture and can be transformed, it could be developed as a platform for studying the role of the collagen‐like adhesins from *Pasteuria* in endospore adhesion.

## INTRODUCTION

Proteins, along with lipids and carbohydrates, make an important component of the surface coat and exosporium of bacterial endospores produced by Gram‐positive bacteria of the Firmicute group and enable them to survive environmentally stressful conditions. The multi‐layered proteinaceous endospore coat that surrounds the core and protects the DNA of the cell, provides mechanical integrity and maintains the endospore's viability in extreme external environments (Driks, 2002; Henriques & Moran, 2000). Several endospore coat proteins serve as enzymes and have been reported to regulate their germination (Foster & Johnstone, 1990; Moir & Cooper, 2016; Paidhungat & Setlow, 2000; Setlow, 2003) while others have also been shown to be involved in adhesion to host surfaces (Henderson et al., 2011; Sánchez et al., 2009).

Endospore proteins have been extensively studied in bacteria belonging to the genus *Bacillus*. The most abundant spore surface protein was discovered in *Bacillus anthracis* and was named as BclA (Bacillus collagen‐like protein of anthracis) for being a collagen‐like glycoprotein (Steichen et al., 2003; Sylvestre et al., 2002, 2003). Localized to the exosporial nap of the *Bacillus* endospores, this glycoprotein has a peptide backbone of ~39 kDa and heavy glycosylation makes the intact protein to have an apparent mass of >250 kDa (Sylvestre et al., 2002). Two other glycoproteins with characteristic collagen‐like (CL) repeats have been reported as ExsJ and ExsH in *B. thuringiensis* and *B. cereus*, respectively (Charlton et al., 1999;García‐Patrone & Tandecarz, 1995; Todd et al., 2003). Both ExsJ and ExsH have been shown to migrate as 205‐kDa species during electrophoresis (García‐Patrone & Tandecarz, 1995; Todd et al., 2003). However, Todd et al. (2003) suggest the existence of the protein ExsJ in two forms: a 205‐kDa multimer and a 70‐kDa monomer. A 58 kDa protein similar to GroEL, a molecular chaperone protein involved in protein folding in several bacteria, has also been found to be present in large quantities in the exosporium of *Bacillus* spp. (Charlton et al., 1999; Redmond et al., 2004). Some other proteins found to be tightly associated with the exosporia in *B. anthracis* include alanine racemase (43 kDa), inosine‐preferring nucleoside hydrolase (33 kDa), ExsF/BxpB (17 kDa), Spore coat protein Z (14 kDa), a CotB homologue (14 kDa), ExsK (10 kDa) and many more (Redmond et al., 2004). Several other exosporium proteins, for example ExsB (26.5 kDa), ExsC (30 kDa), ExsD (66 kDa), ExsE (34 kDa), ExsF (13 kDa) and ExsG (5.4 kDa), have been identified and sequenced in *B. cereus* (Todd et al., 2003).

*Pasteuria penetrans* is an endospore‐forming species of bacterium and a hyperparasite of the economically devastating root‐knot nematode (*Meloidogyne* spp.) pests and is recognized as a potential biological control agent. The restricted host range of *P. penetrans*, that is the ability of a population of *P. penetrans* to adhere to and infect only a particular population of nematodes (Channer & Gowen, 1992; Davies et al., 1988; Stirling, 1985), is a major practical constraint to the successful deployment of *P. penetrans* for the management of rapidly changing heterogeneous populations of nematodes in the field (Liu et al., 2019). Attachment of *Pasteuria* endospores to the cuticle of the host nematodes is an important basis of their host specificity and a determinative step in the infection process. However, the exact molecular mechanisms governing this specificity are unclear. Studies on the cuticle surface coat of plant‐parasitic nematodes have revealed several candidate molecules, for example FAR and mucin‐like proteins, involved in *Pasteuria‐*nematode surface interactions (Phani et al., 2017, 2018). Earlier studies have shown that ageing of the nematode cuticle induces defence responses against bacterial pathogens (Darby et al., 2002; Davies et al., 1991; Gravato‐Nobre & Hodgkin, 2005). Recently, we have demonstrated that plant root‐exudates of host plants reduce the cuticle ageing process thereby recruiting hyperparasitic bacteria and reducing plant‐parasitic nematode burden whereas none host root‐exudates do not (Mohan et al., 2020). Studies over the past several decades have all added to our understanding of the mechanism by which endospores adhere to plant‐parasitic nematode cuticle (Davies, 2009) but as yet our understanding is incomplete.

*Pasteuria* endospores, similar to other endospores, are predominantly composed of proteins and carbohydrates and early studies revealed the presence of at least 15 polypeptide components (Vaid et al., 2002). Previous studies using SDS‐PAGE and exploring different populations of *P. penetrans* had shown variations in banding profiles with more than one unique band for each population (Davies et al., 1992; Davies & Redden, 1997). As these proteins were extracted from the surface of the endospores and exhibited variation between different populations, they could potentially be responsible for the host preferences of these bacteria (Davies, 1994, 2009; Mohan et al., 2001). Proteins glycosylated with *N*‐acetylglucosamine (NAG) have been reported to be associated with the endospore surface and to act as adhesins (Davies & Danks, 1993; Persidis et al., 1991) with the cuticle surface through electrostatic (Afolabi et al., 1995) and/or hydrophobic forces (Davies et al., 1994). Bioinformatics of genome survey sequences of *P. penetrans* show a diverse set of collagen‐like proteins (CLPs) the presence of which appears to be closely related to the BclA protein of *Bacillus* spp. (Davies & Opperman, 2006; Orr et al., 2018; Srivastava et al., 2018). It has been proposed that such BclA‐like proteins constitute the parasporal fibres of the exosporium and may interact with mucin‐like peptides present on the nematode cuticle via a Velcro‐like attachment mechanism (Davies, 2009; Phani et al., 2018). In the cladoceran‐parasitic species, *Pasteuria ramosa*, CLPs have also been previously identified and characterized (McElroy et al., 2011; Mouton et al., 2009). An immunological comparative study between *P. penetrans* and *P. ramosa* has demonstrated that the two species share at least one universally conserved epitope, although the nematode hyperparasite possesses a greater degree of diversity (Schmidt et al., 2008). Phylogenetic studies using a series of housekeeping genes, derived from a genomic survey sequence, have shown *P. penetrans* to be closely related to a saprophytic extremophile *B. haladurans* and the pathogenic and non‐pathogenic strains *B. anthracis* and *B. subtilis*, respectively (Charles et al., 2005), but unfortunately, the insect pathogenic strain *B. thuringiensis*, a similar invertebrate host‐specific parasite and biological control agent of Lepidopteran pests, was not included in this study. This article, therefore, takes a focused approach and characterizes the comparative diversity of endospore CLPs between *P. penetrans* and *B. thuringiensis* with a view to the possibility that, using modern gene editing technologies, *B. thuringiensis* could be developed into a platform/model by which CLPs identified and cloned from *Pasteuria* could be substituted for endospore CLPs in *B. thuringiensis* for gain of function studies.

## MATERIALS AND METHODS

### *Pasteuria* endospores

*Pasteuria penetrans* endospores (isolate Res148) used for characterization experiments were produced in vivo on *Meloidogyne incognita* as per the standard technique (Stirling and Wachtel, 1980) but rather than the roots being dried and macerated, at harvest the roots were frozen overnight and then thawed and *Pasteuria* infected female nematodes were dissected from the roots and homogenized in phosphate buffered saline (PBS) using a 1 ml tissue grinder. Endospores were washed (×3 PBS), counted using a haemocytometer, and an endospore suspension (10^8^ spores/ml in PBS) was stored at −20°C in 500 ml aliquots.

### *Bacillus thuringiensis* cultures and endospores

Three *B. thuringiensis* strains, viz. *B. thuringiensis* str. Al Hakam, *B. thuringiensis* Berliner (ATCC 10792) and a cry‐ mutant of *B. thuringiensis* var. Kurstaki were kindly provided by Dr. A. Bishop. The strains were maintained at the University of Hertfordshire (Hatfield, UK) and stored at −80°C in 40% glycerol in cryovials. The bacteria were sub‐cultured routinely on nutrient agar (Sigma‐Aldrich; N4019) in 9 cm disposable Petri dishes and incubated at 30°C for 24–36 h. For maximum sporulation *Bacillus* strains were cultured in Sporulation Broth (Supplementary Material I(A)) in Nunc™ 6‐well, 3 ml cell culture plates (Thermo Fisher Scientific) and incubated at 37°C at 120 rpm on an orbital shaker. Sporulation was monitored by endospore staining by the Schaeffer‐Fulton technique until at least 95% of the cells had sporulated in 6–8 days (Schaeffer & Fulton, 1933; Smibert & Krieg, 1981; see Supplementary Material I(B)). The broth culture was then centrifuged at 2000 *g* for 10 min. The pellet was washed twice with ice‐cold PBS to remove the nutrient media and finally re‐suspended in 1 ml of sterile distilled water. The suspension was heated to 100°C to induce sporulation in any of the remaining vegetative cells. The heat‐treated suspension was centrifuged at 2000 *g* for 5 min to remove any vegetative debris as supernatant. The purity of the endospores in the pellet was again determined by endospore staining as above. The purified endospores were finally re‐suspended in sterile distilled water. Endospores were counted using a haemocytometer and the count was adjusted to 10^8^ spores/ml. The endospore stock was stored at −20°C in aliquots.

### Antibodies and pre‐immune sera used for immunodetection

The primary polyclonal antibodies were either raised to whole endospores of *Pasteuria* and produced at Rothamsted Research according to Davies et al. (1992) or were obtained from Sigma‐Genosys Ltd., as listed: (i) a polyclonal antibody to the whole endospore of *Pasteuria* (Anti‐PpWS), raised in rabbit (Davies et al., 1992) at Rothamsted Research; (ii) two polyclonal antibodies Col1981 and Col1982, raised in rabbits, to the two custom‐synthetized collagen‐like peptides GTPGTPGPAGPAGPA, and GPQGPQGTQGIQGIQ, respectively (Sigma‐Genosys Ltd.), identified from common CL‐motifs (G–X–Y repeat sequences) found in *Pasteuria* genome survey sequences (Davies & Opperman, 2006). Pre‐bleeds were undertaken before any immunizations and used as pre‐immune sera for negative control to rule out any non‐targeted cross‐reactivity of the other components of the antiserum with the endospore proteins. Following the method described by Davies et al. (1992), antibody working solutions were made to the desired concentration, between 1:500 to 1:2000, depending on the stock antibody concentration in PBST (0.05% v/v Tween in PBS). Anti‐rabbit IgG produced in goat, conjugated either to alkaline phosphatase (A3687; Sigma‐Genosys Ltd.), or to fluorescein isothiocyanate (FITC, F0382; Sigma‐Genosys Ltd.), was used as the secondary antibody.

### Protein extraction from endospores

*Pasteuria* and *Bacillus* endospore suspensions (10^8^ spores/ml) stored at −20°C were thawed at room temperature and centrifuged at 9000 *g* for 2 min. The pellet was re‐suspended in an equal volume of 2× sodium dodecyl sulphate‐polyacrylamide gel electrophoresis (SDS‐PAGE) sample buffer (Supplementary Material I(C)), boiled for 5 min, and then re‐centrifuged at 9000 *g* for 5 min. The supernatant was used as a protein sample for the subsequent protein characterization studies. The protein extracts were quantified by measuring their UV absorbance at 280 nm using Eppendorf BioPhotometer Plus. The final concentration of protein was at least 2 mg/ml for each sample. The extracts were always made in small volumes and stored in 20 μl aliquots at −20°C to avoid repetitive freeze‐thawing.

### Electrophoresis of endospore proteins

Polyacrylamide gel electrophoresis in the presence of SDS (SDS‐PAGE) was done using 12% or 7% (w/v) resolving gel (pH 8·8) and 4% (w/v) stacking gel (pH 6·8). The protein extracts from *Pasteuria* and *Bacillus* endospores (20 μl each) were loaded onto the wells of the stacking gel. Precision Plus Protein™ Standards (Biorad) were used as standard pre‐stained markers of which 5 μl was loaded onto each well. The proteins were allowed to fractionate at a constant voltage of 200 V for about 1.5–2 h. The duration of the electrophoresis run varied as per the molecular weight of the protein under consideration, for example, for proteins larger than 200 kDa, the electrophoresis was run until the 75 kDa protein band in the ladder reached near the bottom of the resolving gel. To visualize the bands separated by SDS‐PAGE, the gels were silver stained using the standard protocol (Wray et al., 1981): the gels were successively placed in fixing solution (50% methanol, 10% acetic acid, 40% de‐ionized water), washing solution (5% methanol, 7% acetic acid, 88% de‐ionized water) and 10% glutaraldehyde, each for 30 min, followed by several subsequent washes in distilled water at least for 4 h. The sensitized gels were then treated with 5 μg/ml dithiothreitol (DTT) for 30 min. Without pouring off DTT, 0.1% silver nitrate solution was added to the gels. After 30 min, the gels were rinsed with distilled water and then with the developer (1 ml 37% formaldehyde per litre of 3% sodium carbonate). The stain was developed by keeping the gels immersed completely in the developer. When the bands developed as desired, the reaction was stopped by adding 2.3 M Citric acid.

### Western blotting and immunodetection

For further characterization of the endospore proteins separated by SDS‐PAGE, the proteins were electro‐blotted onto polyvinylidene fluoride (PVDF) membrane using a Biorad Western blot wet transfer unit at 200 mA constant current for 2 h (Towbin et al., 1979). The tank was kept cool by placing an icepack in it during the transfer. Before setting up the transfer, the PVDF membranes were activated by soaking them in methanol for 15 s, further soaking in distilled water for 2 min and in a blotting buffer for 15 min. The success of the transfer was determined visually by the complete transfer of the pre‐stained ladder. Following the Western Blotting transfer, proteins bound to the PVDF membrane were immunodetected (Davies et al., 1992) as follows. All the incubation and washing steps were performed in small staining trays kept on a rocker at room temperature. The non‐specific binding sites of the membrane were blocked by placing the membrane in a blocking solution (PBST with 1% skimmed milk powder) for 30 min. The blocked membrane was incubated overnight in primary antibody solution (Anti‐PpWS, 1:2000 or Col1981, 1:500 or Col1982, 1:500) at room temperature, followed by subsequent washes in washing solution (PBST). To rule out any non‐specific cross‐reactivity of endospore proteins with rabbit sera, the rabbit pre‐immune antiserum was used in place of the primary antibody as a negative control. The membrane was then incubated in anti‐rabbit IgG (1:1000) for 1 h at room temperature. The proteins specific to the primary antibodies were detected by a chromogenic substrate BCIP (5‐bromo‐4‐chloro‐3‐indolyl‐phosphate). A solution of disodium salt of BCIP (2 mg/ml) was freshly prepared in diethanolamine (DEA) buffer. The membrane was placed in the substrate solution until blue‐coloured bands developed. Once the desired colour developed, the membrane was washed in distilled water for about 10 min and allowed to air‐dry. The blue bands on the membrane were observed. These bands corresponded to the endospore proteins recognized by the primary antibodies used. See Supplementary Material I(D) for the list and composition of all the reagents used in Western blot analysis.

### Detection of glycoproteins by glycoprotein staining

The endospore protein extracts were electrophoresed and blotted onto the PVDF membrane as described above. The proteins were stained using the Pierce™ Glycoprotein Staining Kit (Thermo Fisher Scientific) for the detection of carbohydrate moieties conjugated to any glycoproteins present in the sample. All the procedures for staining were performed as per the manufacturer's instructions; incubation and washing steps were done in small staining trays kept on a rocker at room temperature. The membrane was observed for the presence of magenta‐coloured bands of glycoproteins.

### Detection of glycoproteins by lectin blotting

The endospore protein extracts were separated by SDS‐PAGE and blotted on to PVDF membrane using the standard Western blotting technique as described previously. Biotinylated wheat germ agglutinin (WGA) (5 mg/ml in 10 mM HEPES, 0.15 M NaCl, pH 7.5, 0.08% sodium azide, 0.1 mM Ca^2+^ obtained from Vector Labs) was then used to detect any glycoproteins with NAG as their glycoconjugates. All the washing and incubation steps were done as follows in small staining trays kept on a rocker at room temperature. The non‐specific binding sites of the PVDF membrane were blocked by placing the membrane in 1% bovine serum albumin (BSA) in TTBS (Tris‐buffered saline with 0.1% Tween) for 30 min. The blocked membrane was transferred to 5 μg/ml biotinylated WGA solution in TTBS and incubated for 1 h. The membrane was washed thrice in TTBS over 15 min and, thereafter, incubated in Vectastain® ABC‐AP reagent (AK‐5000; Vector Laboratories Ltd.) for 30 min. The ABC‐AP reagent was prepared as per the manufacturer's instructions (see Supplementary Material I(E)). The membrane was again washed thrice in TTBS over 15 min, followed by washing once in 100 mM Tris, pH 9.5 buffer. Finally, the membrane was placed in a freshly prepared substrate solution containing 2 mg/ml BCIP in 10% DEA buffer until the appearance of blue bands that corresponded to the glycoproteins which have NAG as their glycoconjugates. Once the desired colour developed, the membrane was washed in distilled water for about 10 min and allowed to air‐dry.

### Detection of collagens in endospore protein extracts

To digest any collagens, present in the heterogeneous protein mixture extracted from *Pasteuria* and *Bacillus* endospores, the protein extract was treated with collagenase enzyme in PBS‐Ca^2+^ buffer (1500 U/ml stock of C0773 from *Clostridium histolyticum*; Sigma‐Aldrich). Before the enzyme treatment, the protein extracts were desalted and precipitated using acetone to remove any salts or buffer components that could interfere with the enzyme activity. The protein extracts were diluted with sterile H_2_O, loaded on to Vivaspin® 2 centrifugal concentrators (Viva products, Inc.) and centrifuged at 4000 *g* for 15 min at 4°C in a Heraeus Labofuge 400R centrifuge (Thermo Fisher). The flow‐through was discarded and the retentate was topped up to 1 ml with sterile H_2_O. The steps were repeated twice. The final retentate was subjected to acetone precipitation to remove any SDS micelles still present. Ice‐cold acetone was added to the Vivaspin‐desalted extracts in a 4:1 ratio. The acetone‐protein mixture was incubated at −20°C for 1 h and then centrifuged at 13,000 *g* for 10 min. The supernatant was removed carefully, leaving a volume equivalent to about half of the initial volume of the Vivaspin‐desalted extract used for precipitation. Any traces of acetone in the precipitated protein were allowed to evaporate at room temperature. The protein precipitate was re‐dissolved in sterile PBS. For collagenase treatment, 6 μl of each protein extract was mixed with 4 μl of collagenase enzyme stock (1500 U/ml) in a 25 μl‐tube and incubated at 37°C for 2 h. Collagenase‐treated protein extracts (10 μl of each) were diluted to an equal volume of 2× SDS‐PAGE sample buffer. The SDS‐PAGE and Western Blots were performed as described previously. The untreated protein samples were run simultaneously as controls. The two anti‐collagen antibodies Col1981 and Col1982 were used to detect the presence of two specific collagen‐like peptides as described earlier. Collagens, with NAG as their glycoconjugate, were detected by a lectin blot as previously. The bands obtained for untreated protein extracts were compared to those obtained for collagenase‐treated extracts. A shift in the location of a band to a lower molecular weight marker or a missing band in collagenase‐treated extracts would indicate a collagen or CL peptide. Gelatin (2 mg/ml stock), mixed with an equal volume of 2× sample buffer, was used as a positive control.

### Immunolocalization of surface epitopes on *P. penetrans* and *B. thuringiensis* endospores

The wells of multi‐well slides were coated with 1:10 dilution of 0.1% Poly‐l‐lysine (P8920; Sigma Aldrich) in deionized water and left to dry. Once dry, the wells were coated with 10 μl of 10^5^ endospores/ml suspension of either *P. penetrans* or *B. thuringiensis* Al Hakam. After the suspension had air dried, each well was overlayered with 10 μl of PBST with 2% BSA and incubated for 1 h at room temperature to block the non‐specific binding sites. This step was one of the critical steps in reducing background fluorescence. Each well was then overlayered with 10 μl of a 1:50 dilution of either Anti‐PpWS or Col1982 in PBST. The slides were incubated in a moist chamber for 2 h at room temperature, followed by three subsequent gentle washes in PBST to remove the excessive unbound antibodies. The wells were then covered with 10 μl of a 1:50 dilution of the FITC‐labelled anti‐rabbit IgG in PBST. The slides were incubated in dark and moist chamber for 1 h at room temperature, followed by three subsequent gentle washes in PBST to remove the excessive unbound antibodies. A coverslip with a drop of Citifluor mounting fluid (Citifluor Ltd.) was mounted on each well to prevent photo‐bleaching of the fluorescent samples. The coverslips were sealed to prevent drying. The prepared slides were observed under microscope as freshly as possible. When needed, the slides were stored at 4°C in moist and dark chamber. The immune‐stained endospore slides were observed first under the 40× objective and then under the oil immersion objective (100×) using the B1‐E bandpass emission filter of Nikon Optiphot‐2 Fluorescent microscope.

### Immunolocalization of surface exposed NAG residues on *Pasteuria* endospores

*Pasteuria penetrans* endospores were attached to slides coated with poly‐l‐lysine, and the non‐specific binding sites were blocked as described above. The endospores in each well of the slide were overlayered with 10 μl of FITC‐labelled WGA (5 mg/ml in 10 mM HEPES, 0.15 M NaCl, pH 7.5, 0.08% sodium azide, 0.1 mM Ca^2+^ as obtained from Vector Labs) and incubated in dark and moist chamber at room temperature for 2 h. The unbound WGA was removed by gently washing the wells thrice with PBST. The slides were mounted and as described previously.

To identify if any of the epitopes recognized by the Anti‐PpWS or Col1982 were glycosylated with NAG, the *P. penetrans* or *B. thuringiensis* Al Hakam endospores were pre‐treated with unconjugated WGA, followed by indirect immunofluorescence using either Anti‐PpWS or Col1982 as the primary antibody, and FITC‐labelled anti‐rabbit IgG as the secondary antibody. The unconjugated WGA was obtained in a lyophilized form and was diluted to 5 mg/ml in 10 mM HEPES buffered saline, pH 8.5, 0.1 mM CaCl_2_. The endospores adhering to the poly‐l‐Lysine‐coated wells of a multi‐well slide were overlayered with unconjugated WGA and incubated for 1 h in a moist chamber. The aim was to let WGA bind to all the surface‐exposed NAG residues. The unbound WGA was removed by washing the wells thrice gently with PBST. After treating the endospores with WGA, each well was overlayered with 10 μl of a 1:50 dilution of either Anti‐PpWS or Col1982 in PBST. The slides were incubated in a moist chamber for 2 h at room temperature, followed by three subsequent gentle washes in PBST to remove the excessive unbound antibodies. The wells were then covered with 10 μl of a 1:50 dilution of the FITC‐labelled anti‐rabbit IgG in PBST. The slides were mounted in Citifluor and examined as described previously.

### Imaging and quantification of fluorescence

The fluorescent microscopy images along with their corresponding bright field images were captured at 1000× magnification using a Nikon D5200 DSLR camera attached to the microscope. The resulting fluorescent images were then analysed using Fiji, an image processing and analysis software tool; the fluorescence was quantified and measured in terms of corrected total cell fluorescence (CTCF) (Schindelin et al., 2012). The mean CTCF values for each treatment of bacterial endospores were compared using one‐way analysis of variance (ANOVA) and Tukey's post hoc test to find the means that significantly differed from each other.

### Nematode juveniles and *Pasteuria* endospores for attachment assays

Freshly hatched second stage juveniles (J2) of root knot nematodes (RKN), *M. incognita*, were used for the attachment assays. To obtain a fresh J2 culture, the following protocol was followed. Aubergine (*Solanum melongena*) plants growing in pots for 28 days were uprooted and the roots were washed gently with tap water; egg masses of root knot nematodes were handpicked using forceps and collected in a small volume of sterile distilled water in a petri dish and incubated at room temperature (25 ± 2°C) overnight. The freshly hatched J2s were concentrated. Excess water was carefully removed using a pipette. The nematode suspension was swirled gently to make it homogeneous. The nematode count was adjusted to about 100 J2/10 μl of suspension.

*Pasteuria* endospores were obtained by dissecting infected female root‐knot nematodes obtained from cowpea (*Vigna unguiculata*). The spores were washed repeatedly in sterile distilled water and pelleted by centrifugation (1000 *g* for 10 min). The final concentrated spores were re‐suspended in PBS and the spore count was adjusted to 10^5^ spores/ml using a haemocytometer.

### Pre‐treatment of *Pasteuria* endospores and nematode juveniles for attachment assays

Table 1 lists all the chemicals used for the treatments and summarizes their dilutions and the optimum temperature at which the treated endospores/ juveniles were incubated. For each endospore treatment, 5 μl of homogeneous endospore suspension (~500 spores) was concentrated at the bottom of a sterile 0.2 ml PCR tube by centrifugation (1000 *g* for 10 min at room temperature). The supernatant was removed; the pellet was re‐suspended in 50 μl of the diluted treatment chemical (antibody/enzyme/lectin) and incubated at optimum temperatures for 1 h. For the treatment of nematode juveniles, 10 μl of homogeneous J2 suspension (~100 J2) was concentrated at the bottom of a sterile 0.2 ml PCR tubes by centrifugation (1000 *g* for 3 min at room temperature). The treatments were done as directed above for endospores. After washing, the treated J2s were resuspended in 10 μl of PBS. Control sets of untreated endospores and nematode juveniles were incubated in PBS at room temperature prior to the attachment assay. The treated and untreated endospores/juveniles were then washed thrice with PBST (0.05% v/v PBS with Tween‐80) by repeated centrifugation and removing the supernatant each time. The final pellet of endospore and juveniles were re‐suspended in 5 and 10 μl of PBS, respectively.

**TABLE 1:** Treatments given to *Pasteuria* endospores and RKN juveniles prior to attachment assays

Treatments	Dilutions	Diluent	Incubation	Treatment role
Pre‐incubation of *Pasteuria* endospores with Col1981	1:50	PBS	25 ± 2°C for 1 h	To block the surface‐exposed collagens of *Pasteuria* endospores
	1:500			
	1:1000			
Pre‐incubation of *Pasteuria* endospores with Col1982	1:50	PBS	25 ± 2°C for 1 h	To block the surface‐exposed collagens of *Pasteuria* endospores
	1:500			
	1:1000			
Pre‐incubation of *Pasteuria* endospores and RKN J2 with Collagenase	750 U/ml	PBS‐Ca^2+^	37°C for 1 h	To digest the surface‐exposed collagens of *Pasteuria* endospores and on J2 cuticle
	375 U/ml			
	250 U/ml			
Pre‐incubation of *Pasteuria* endospores and RKN J2 with *N*‐acetylglucosaminidase (NAGase)	50 U/ml	PBS	37°C for 1 h	To digest the surface‐exposed NAG residues of *Pasteuria* endospores and on J2 cuticle
	25 U/ml			
Pre‐incubation of *Pasteuria* endospores and RKN J2 with WGA	1:50	PBS	25 ± 2°C for 1 h	To block the surface‐exposed NAG residues of *Pasteuria* endospores and on J2 cuticle
	1:500			
	1:1000			

Abbreviations: PBS, phopshate buffered saline; RKN, root knot nematodes; WGA, wheat germ agglutinin.

### Attachment bioassays

To each tube containing 5 μl of treated endospore suspension (~500 spores), 10 μl of untreated J2 suspension (~100 J2) was added; conversely, untreated endospores (5 μl) were added to treated J2 suspension (10 μl). A control assay was done with 5 μl of untreated endospore suspension with 10 μl of untreated J2 suspension. The nematode‐*Pasteuria* mixture was centrifuged at 2000 *g* for 5 min. The mixture was left undisturbed at 4°C overnight, and attachment was observed the next morning. For each treatment, 20 nematode juveniles were randomly selected and observed under light microscope (400× magnification). The number of spores attached per juvenile was counted. The average numbers of endospores attached after the different treatments at different concentrations were compared using one‐way and two‐way ANOVA and Tukey's post hoc test to find the effect of treatments that significantly differed from each other.

## RESULTS

### Purification of endospores of *Bacillus* spp.

Maximum sporulation was observed on the eighth day after inoculation. The endospores obtained and purified from the sporulation broth were at least 98% pure as observed by endospore staining.

### SDS‐PAGE of protein extracts from *Bacillus* and *Pasteuria* endospores

**FIGURE 1:**
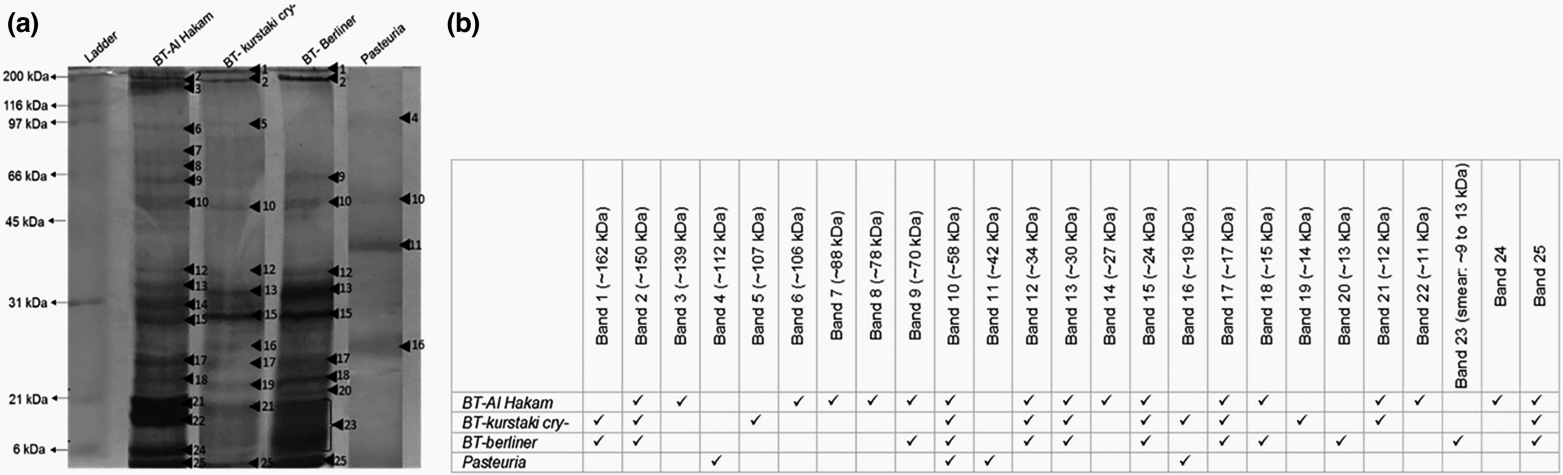
Protein profiles of endospore protein extracts separated by SDS‐PAGE and stained by silver stain (12% resolving, 4% stacking gels; electrophoresis at 200 V for 45 min). (a) Image of the silver‐stained gel (prominent bands marked as arrowheads, numbered in decreasing order of their molecular weights); (b) tabulated representation of the occurrence of the bands in different bacterial endospore samples (band numbers are same as numbered in the image). See Supplementary Material A (Table 1) for calculations to estimate molecular weight. SDS‐PAGE, sodium dodecyl sulphate‐polyacrylamide gel electrophoresis

Silver stain detected at least four bands in *Pasteuria* endospore extracts. An ~58 kDa band was commonly observed in all the bacterial strains while an ~19 kDa band was common in *P. penetrans* and *B. thuringiensis* Kurstaki strain. Two distinct bands (~112, ~42 kDa) were peculiar to *P. penetrans* (Figure 1a,b). See the Supplementary Material for calculations and graphs used for the estimation of molecular weights.

### Immunodetection of endospore proteins using Anti‐PpWS

**FIGURE 2:**
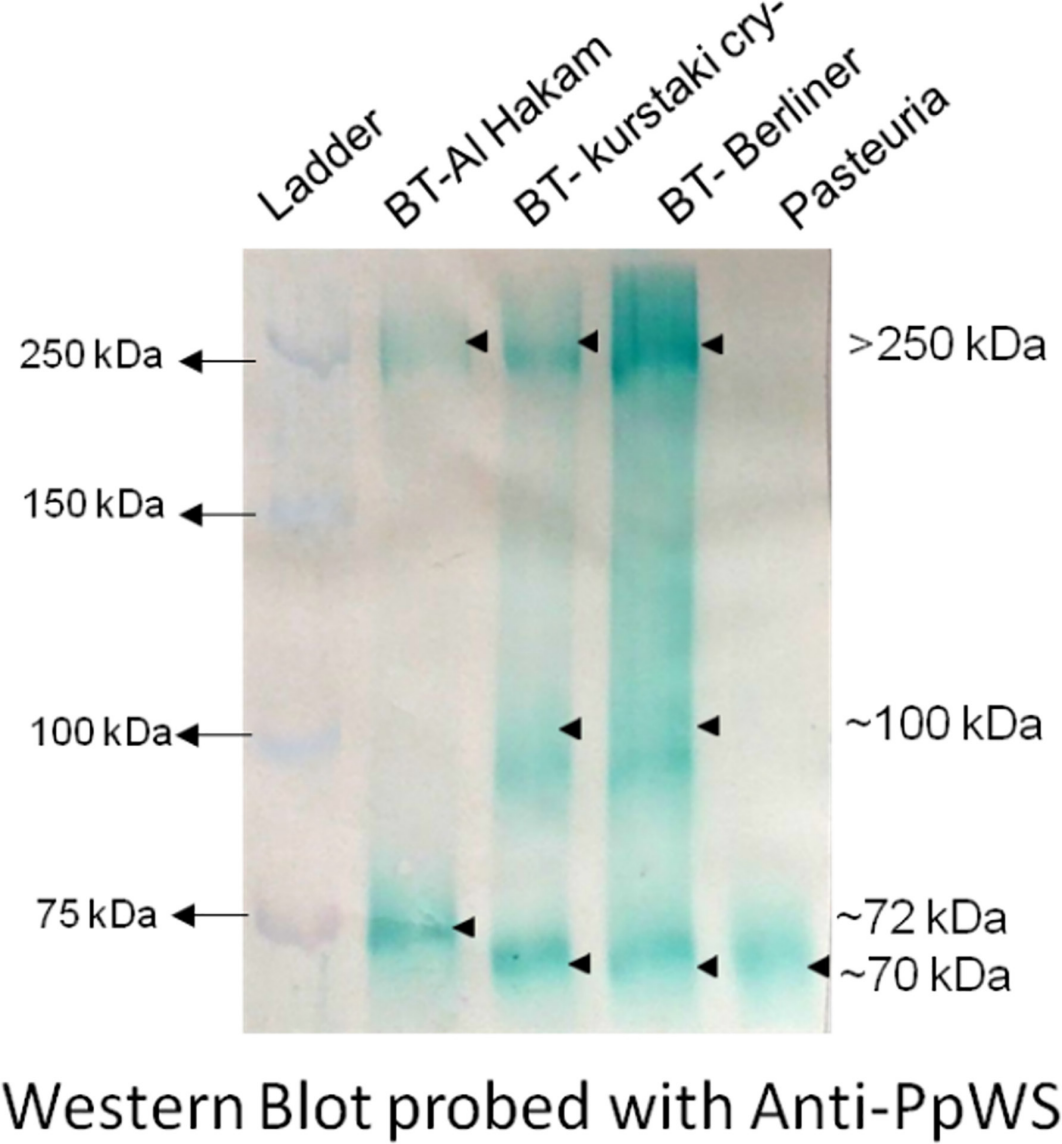
Immunodetection of endospore protein extracts from three strains of *Bacillus thuringiensis* and *Pasteuria penetrans* Res148 using anti‐PpWS. See Supplementary Material F (Table 2) for calculations to estimate molecular weight

The proteins on SDS‐PAGE gels were successfully transferred to PVDF membranes by Western blotting as evident from the complete transfer of pre‐stained protein markers from the gel onto the membrane. Probing the Western blot with Anti‐PpWS antibody revealed two bands in *B. thuringiensis* Al Hakam, three bands each in *B. thuringiensis* Berliner and *B. thuringiensis* Kurstaki, while just one band in *P. penetrans* Res148 (Figure 2). A band of ≥250 kDa was common to all *B. thuringiensis* strains but was not visible in *Pasteuria*. An ~99 kDa band was observed in Kurstaki and Berliner strains of *B. thuringiensis* but not in Al Hakam, while an ~75 kDa band was only observed in Al Hakam. An ~72 kDa band was common to *Pasteuria* and *B. thuringiensis* Kurstaki cry‐ and Berliner.

### Immunodetection of endospore collagens using Col1981/Col1982

**FIGURE 3:**
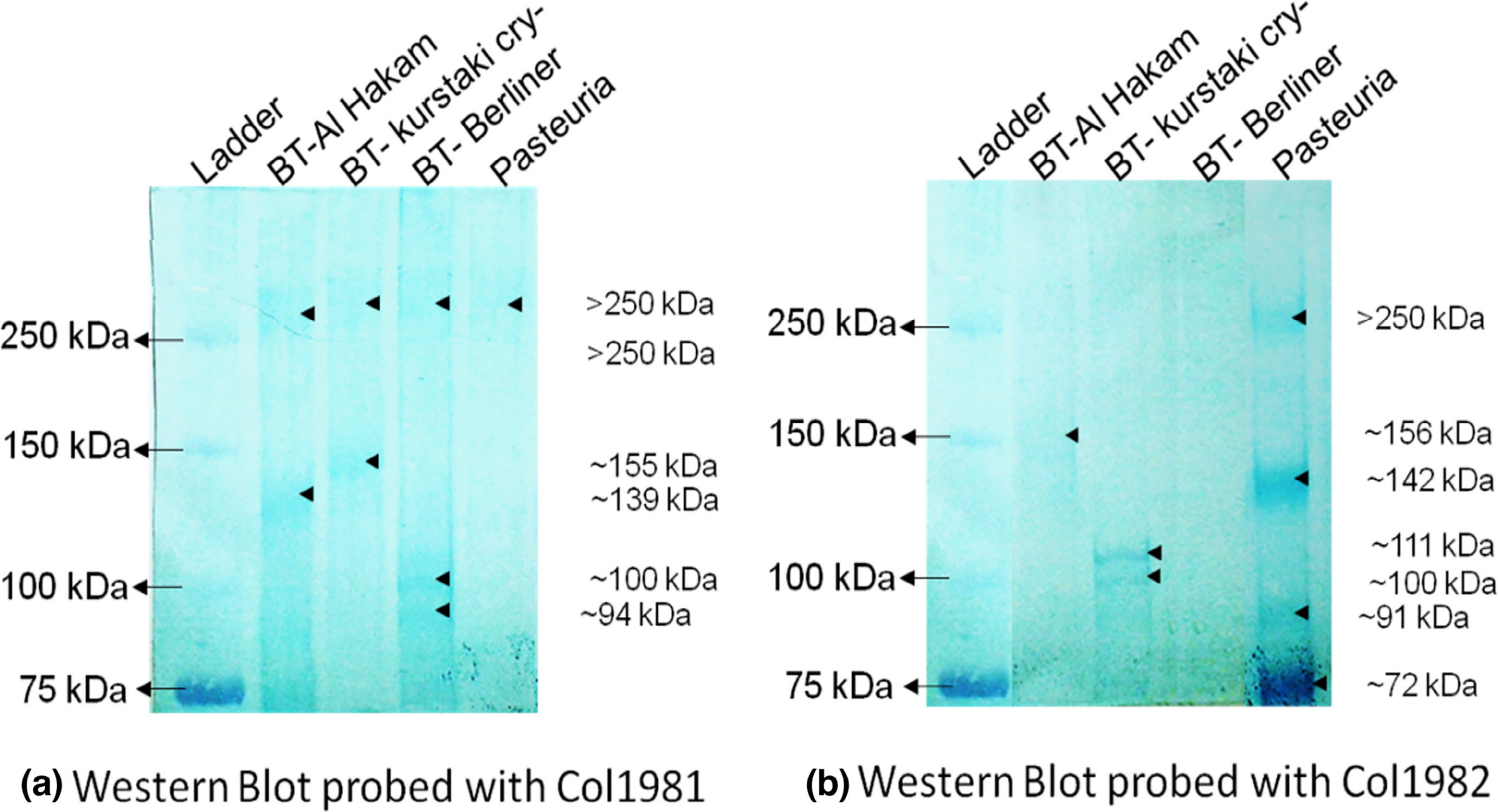
Immunodetection of collagen‐like proteins in the endospore protein extracts from three strains of *Bacillus thuringiensis* and *Pasteuria penetrans* Res148 using Col1981 and col 1982. See Supplementary Material F (Tables 3 and 4) for calculations to estimate molecular weight

When probed with the Col1981 antibody, endospore extracts revealed one band of >250 kDa in each of the bacterial samples (Figure 3a); this band migrated slightly less in *Pasteuria*, *B. thuringiensis* Berliner and *B. thuringiensis* Kurstaki than in *B. thuringiensis* Al Hakam. No other band was visible in *Pasteuria* extract, while there were two additional bands in *B. thuringiensis* Berliner (~103, ~94 kDa) and one band each in *B. thuringiensis* Al Hakam (~139 kDa) and *B. thuringiensis* Kurstaki (155 kDa).

The Col1982 antibody recognized a ≥250 kDa band and three other protein bands in *Pasteuria* (~142, ~91, ~72 kDa), one protein band in *B. thuringiensis* Al Hakam (~156 kDa), two bands in *B. thuringiensis* Kurstaki (~111, ~103 kDa) and no bands in *B. thuringiensis* Berliner (Figure 3b).

### Detection of glycoproteins

**FIGURE 4:**
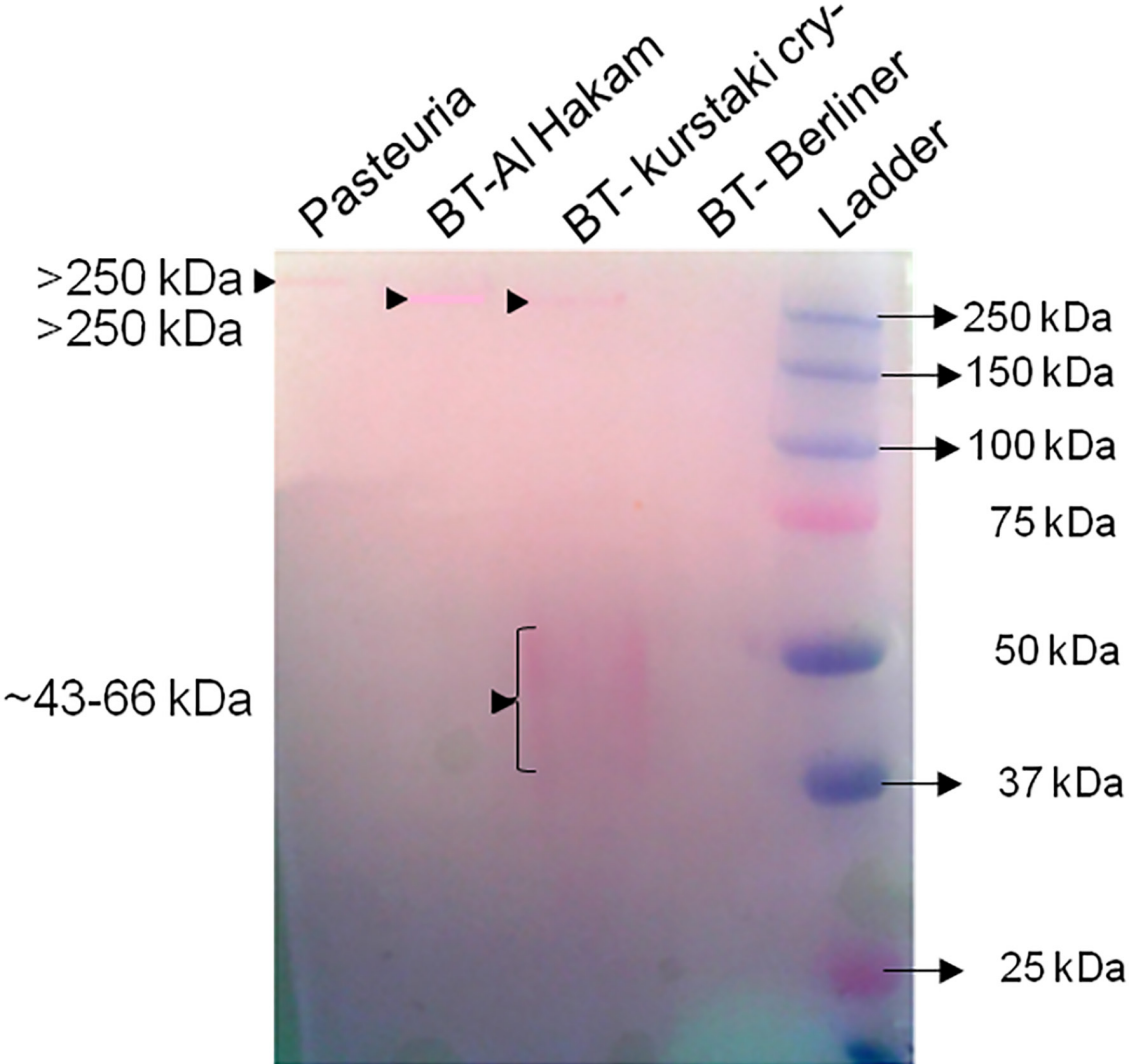
Detection of glycoproteins in endospore protein extracts: SDS‐PAGE gel stained with Thermo Scientific Pierce™ glycoprotein staining kit. See Supplementary Material F (Table 5) for calculations to estimate molecular weight. SDS‐PAGE, sodium dodecyl sulphate‐polyacrylamide gel electrophoresis

Staining of SDS‐PAGE gels with the Thermo Scientific Pierce™ Glycoprotein Staining Kit revealed a band of more than 250 kDa in *Pasteuria* endospore protein extract (Figure 4). Similar higher molecular weight bands were observed in two of the *B. thuringiensis* strains, namely, *B. thuringiensis* Al Hakam and *B. thuringiensis* Kurstaki. Additionally, a smear of about 43–66 kDa was also visible in *B. thuringiensis* Kurstaki. No glycoprotein was detected in *B. thuringiensis* Berliner.

**FIGURE 5:**
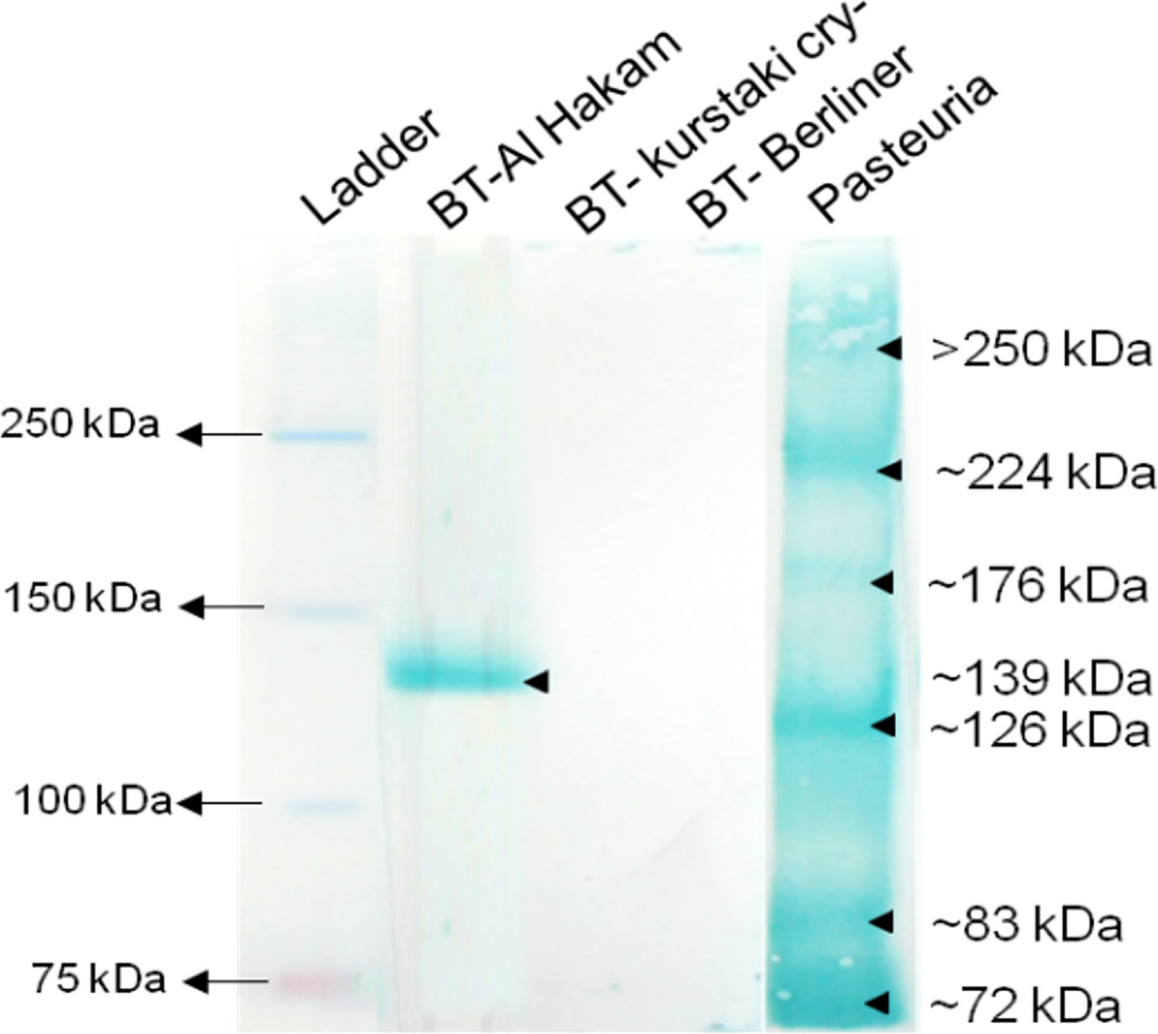
Western blot probed with WGA for the detection of glycoproteins with NAG as glycoconjugates. See Supplementary Material F (Table 6) for calculations to estimate molecular weight. NAG, *N*‐acetylglucosamine; WGA, wheat germ agglutinin

Probing the Western blots of endospore extracts with WGA (a lectin that detects NAG) revealed a single band of about 139 kDa in *B. thuringiensis* Al Hakam, while no bands in the other *B. thuringiensis* strains (Figure 5). In *P. penetrans* Res 148 endospore extracts, at least six bands were distinctly visible (>250, ~224, ~176, ~126, ~83, ~69 kDa). These results indicated that some of the polypeptides resolved from *B. thuringiensis* and *P. penetrans* endospores are glycosylated with NAG.

### Detection of collagens after collagenase treatment of protein extracts

**FIGURE 6:**
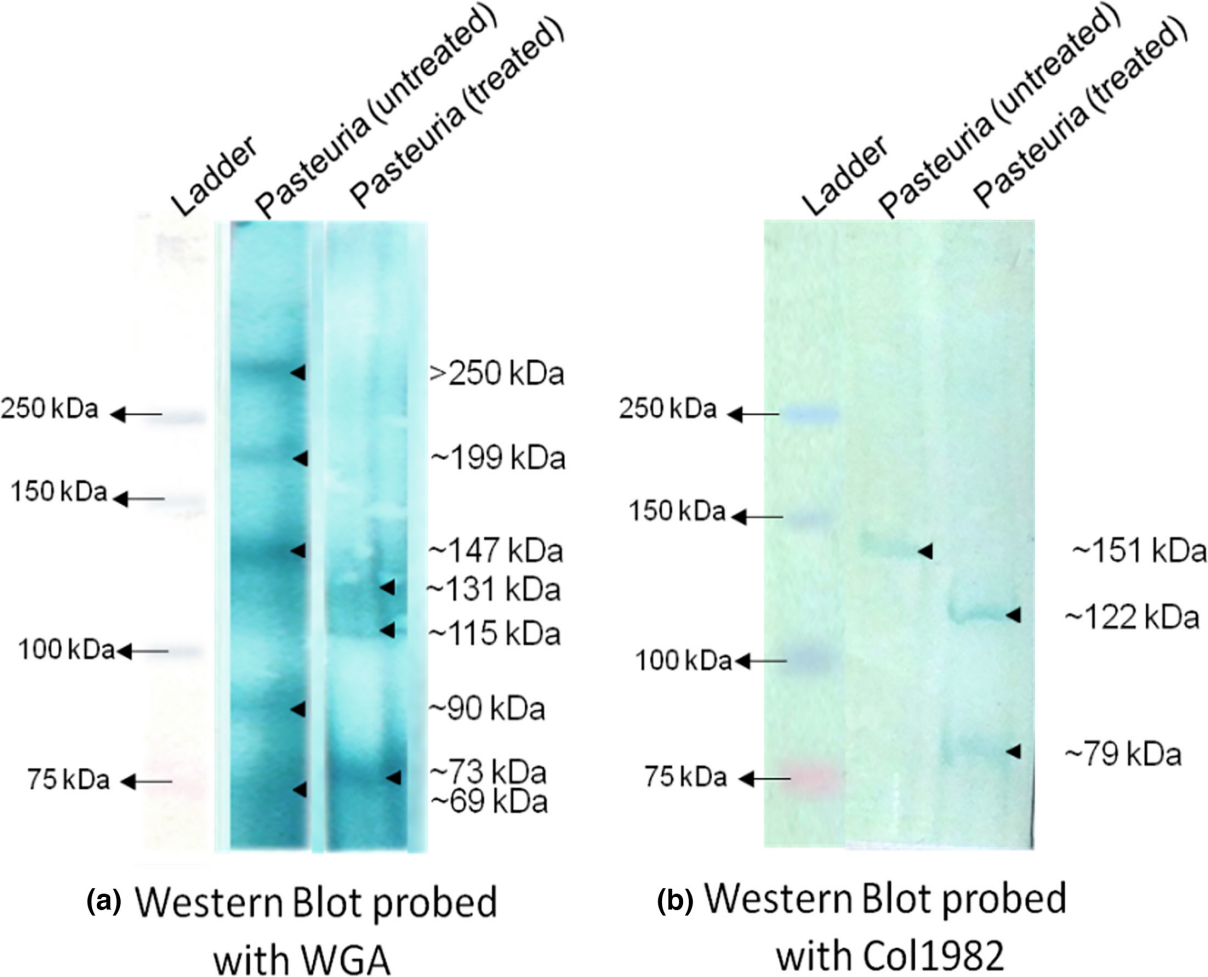
Detection of collagens after 6 h of collagenase treatment. See Supplementary Material F (Tables 7 and 8) for calculations to estimate molecular weight

The success of collagenase treatment was confirmed by a visual shift of higher molecular weights bands to lower molecular weight in a gelatin sample (data not shown). When the protein extracts from *Pasteuria* endospores were treated with collagenase for 2 h, no major differences were observed in the banding pattern of the WGA‐probed Western blot as compared with the untreated samples (data not shown). When the protein extracts were treated with collagenase enzyme for 6 h, the number of bands recognized by WGA decreased from five bands in untreated protein extract (>250, ~199, ~147, ~90, 69 kDa) to three bands in the treated extract (~131, ~115, ~73 kDa) (Figure 6a). This indicates that some of the proteins/peptides were digested, partially or completely, and the proteins/peptides or peptide fragments detected on the blot were the ones that had NAG attached to them as a glycoconjugate. When the Western blot of the same samples (6 h collagenase‐treated) was probed with Col1982, it produced a single band of about 151 kDa in untreated protein sample whereas two distinct bands at about 122 and 79 kDa in collagenase‐treated protein sample of *Pasteuria* endospores (Figure 6b).

### Immunolocalization of surface epitopes using Anti‐PpWS and Col1982

**FIGURE 7:**
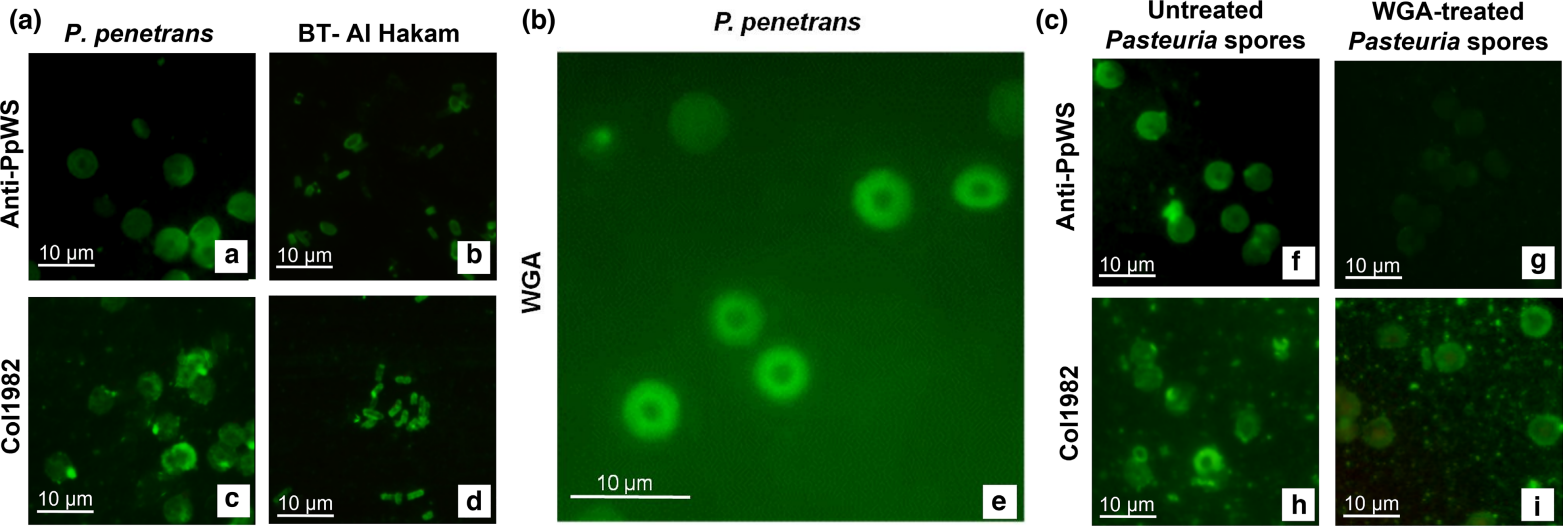
Representative fluorescent micrographs illustrating the immunolocalization of immunodominant epitopes on the surface of *Pasteuria penetrans* Res 148 and *Bacillus thuringiensis* Al Hakam endospores. The images were captured at 1000× magnification and digitally zoomed to 2× at desired focal point. (Total magnification = 2000×). The scale bar measures 10 μm. (A) Immunolocalization of common surface epitopes on the endospores of *P. penetrans* and *B. thuringiensis* Al Hakam when probed with polyclonal antibodies raised to whole endospores of *Pasteuria* Anti‐PpWS (a, b) and collagen‐specific polyclonal antibodies Col1982 (c, d), followed by indirect immunofluorescence using FITC‐labelled anti‐IgG. (B) Immunolocalization of surface‐exposed *N*‐acetylglucosamine residues by direct immunofluorescence using FITC labelled wheat germ agglutinin (e). (C) Reduction in recognition of Anti‐PpWS (f, g) and Col1982 (h, i) antibodies when *Pasteuria* endospores were pre‐treated with unconjugated wheat germ agglutinin. (FITC, fluorescein isothiocyanate)

Both Anti‐PpWS and Col1982 were able to recognize endospore surface epitopes of *P. penetrans* as anticipated. More interestingly, the two antibodies also recognized the epitopes present on the surface of *B. thuringiensis* endospores. At a 2000× magnification (1000× microscopic magnification; 2× digital zoom in), it was evident that Anti‐PpWS did not bind to the central body of the *Pasteuria* endospores (Figure 7A). Two concentric circles of fluorescence were observed, one just outlining the central body and the other one on the periphery of the endospores; these two concentric layers recognized by Anti‐PpWS were separated by a very fine line of non‐fluorescent layer (Figure 7A[a]). Similarly, in case of *B. thuringiensis* endospores probed with Anti‐PpWS, the surface epitopes were recognized more at the periphery of the endospores than at the center (Figure 7A[b]). Collagen‐specific antibody Col1982 recognized more epitopes on the periphery of both *Pasteuria* and *B. thuringiensis* endospores (Figure 7A[c,d]).

### Immunolocalization of surface exposed NAG residues

When the endospores were probed with WGA, a lectin that specifically binds to NAG, the presence of NAG residues distributed in a definite pattern on the surface of the *P. penetrans* endospores was evident by strong fluorescence (Figure 7B). There was a clear pattern in which WGA recognized the endospores as observed in case of Anti‐PpWS probing; there were two concentric circles of fluorescence observed, one outlining the central body and the other one on the periphery of the endospore. The inner fluorescent layer was much more intense than the outer layer. When the endospores were pre‐treated with unconjugated WGA followed by probing them with Anti‐PpWS, there was a highly significant decrease based on CTCF values (*p =* 3.09e^−10^; (Schindelin et al., 2012) in the recognition of the antibody (Figure 7C). Similarly, when WGA‐treated endospores were probed with Col1982, there was again a marked decrease based on CTCF values (*p =* 0.0289; Schindelin et al., 2012) in the recognition of the endospores as compared to the untreated endospores.

### Effect of different chemical treatments on the adhesive abilities of *Pasteuria* endospores

**FIGURE 8:**
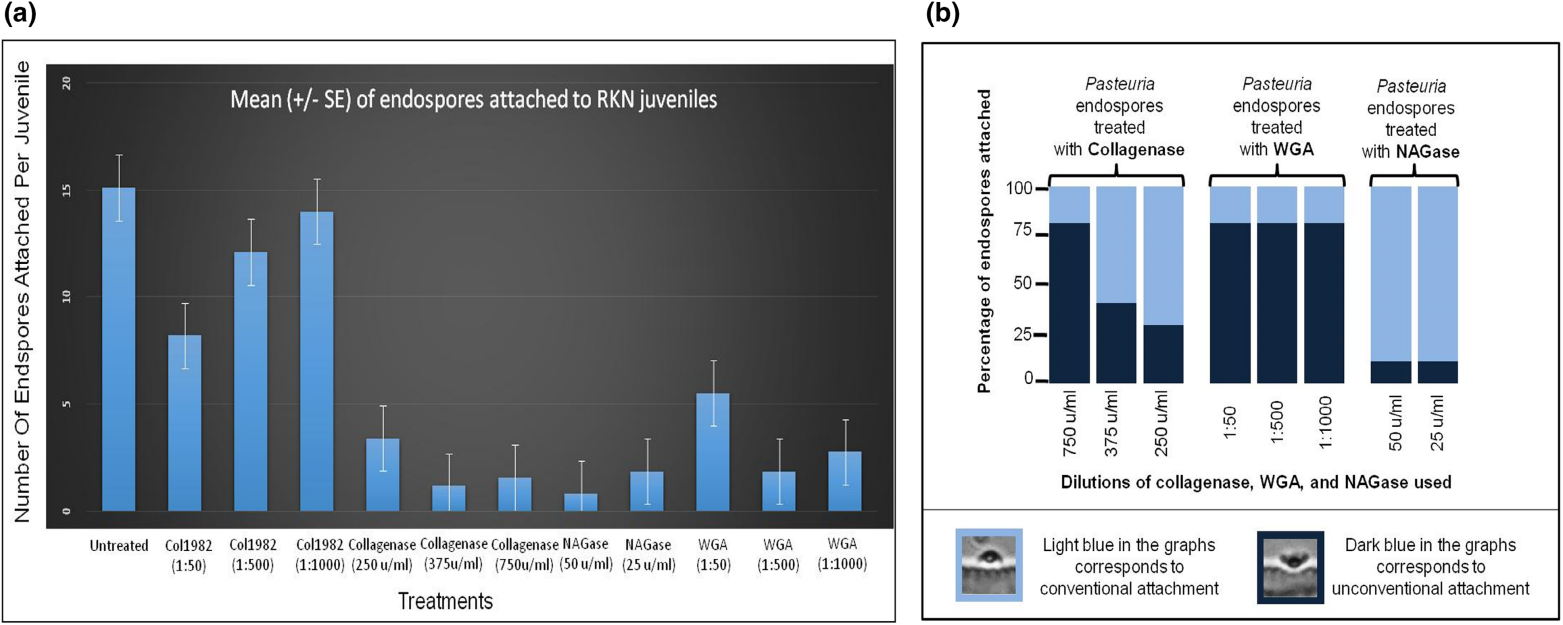
(a) Effects of different treatments of *Pasteuria* endospores on their in vitro attachment to the juveniles of root knot nematodes. Water and phosphate buffer saline (PBS) were used as control treatments; (b) percentage of conventional and unconventional type of endospore attachment observed when *Pasteuria* endospores pre‐treated with collagenase, WGA and NAGase were allowed to interact with untreated RKN juveniles. RKN, root knot nematodes; WGA, wheat germ agglutinin

1. Attachment of untreated endospores: When a set of untreated *Pasteuria* endospores were allowed to interact with a set of freshly hatched second stage juveniles of root knot nematodes, as many as 24 endospores per J2 were observed to attach in the 20 J2s observed. On an average the number of endospores attached per J2 was 15.1 ± 1.05. All the endospores attached in the conventional manner, that is, the concave surface of the skirt‐like structure of the exosporium was in contact with the nematode cuticle. In general, the endospores did not show preference to any region of the nematode body, they attached equally well to the anterior, middle and posterior regions of the juveniles.
2. Effect of collagen blocking/digestion on endospore attachment: *Pasteuria* endospores pre‐incubated with the collagen‐specific antibody Col1982 showed marked concentration‐dependent reduction in their ability to attach to the healthy root knot juveniles as compared to the untreated endospores (*F*_1, 38_ = 76.72, *p* = 1.19e‐10 at 1:50 dilution of Col1981; *F*_1, 38_ = 33.08, *p* = 1.25e‐06 at 1:50 dilution of Col1982) (Figure 8a). Even at the least concentrations of Col1982 (1:1000), the number of endospores attached per J2 reduced by 7.3%. The most effective dilution of Col1982 (1:50) caused a marked decrease of 45.7% in Col1982‐treated endospores. When the endospores were treated with collagenase enzyme, all the three dilutions were very effective in reducing the attachment (*F*_1, 38_ = 90.53, *p* = 1.34e‐11 at 250 U/ml collagenase), percentage reduction compared to the untreated control being 89.7%, 92.4% and 77.5% for 750, 375 and 250 U/ml collagenase treatments, respectively (Figure 8a).
3. Effect of NAG blocking/digestion on endospore attachment: When endospores were pre‐incubated with WGA, the number of endospores attached per juvenile was reduced by 94.7% at 1:50 dilution, the least dilution (1:1000) showing a reduction of 63.6% (*F*_1, 38_ = 174.6, *p* = 8.73e‐16 at 1:50 dilution of WGA*; F*_1, 38_ = 61.79, *p* = 1.74e‐09 at 1:1000 dilution of WGA. Treatment of the endospores with the enzyme *N‐*acetylglucosaminidase (NAGase) resulted in 81.8% reduction in the number of endospores attached per J2 at 25 U/ml of NAGase (*F*_1, 38_ = 120.4, *p* = 2.44e‐13 for endospores*; F*_1, 38_ = 92.31, *p* = 1.03e‐11 for J2) (Figure 8a).
4. Unconventional attachment of pre‐treated *Pasteuria* endospores: A unique observation was observed in the orientation of the attached *Pasteuria* endospores to nematode juveniles. *Pasteuria* endospores commonly attach to the nematode cuticle with their concave surface. Here, while most of the untreated endospores attached to nematode juveniles in a conventional manner, endospores pre‐treated with collagenase, WGA and NAGase not only caused a reduction in the number of endospores attached per juvenile but also triggered the endospores to attach unconventionally, through their convex surface (Supplementary Material Figure S1). The percentage of unconventionally attached endospores increased to 80% for collagenase‐treated endospores and for WGA‐treated spores, and 40% for NAGase‐treated spores (Figure 8b).

## DISCUSSION

We have demonstrated that both *P. penetrans* and *B. thuringiensis* share collagen‐like glycoproteins that are likely associated with the surfaces of their endospores. The obligate nature of the *Pasteuria* group makes studying the role of the endospore surface adhesins difficult. Endospores of *B. thuringiensis* do not naturally adhere to second‐stage juveniles of root‐knot nematodes, and therefore, the new gene editing capabilities that would allow endospore surface expressed collagen‐like adhesins in *B. thuringiensis* to be replaced with those from *P. penetrans* would allow collagen‐like gain of function studies to be conducted using *B. thuringiensis* as an easily cultured model bacterium. Our novel approach of comparing the surface epitopes of *Pasteuria* and *Bacillus* endospores could help in improving the understanding of endospore biochemistry and antigenic determinants of the surface of the *Pasteuria* exosporium. Furthermore, we provide experimental evidence for the role of NAG glycosylated CLPs in the initial adhesion of endospores to the nematode cuticle.

The development of *P. penetrans* as a biological control agent for plant‐parasitic nematodes is greatly inhibited by the bacterium's obligate nature and host range; understanding the nature of the endospore surface is fundamental for its successful deployment as a control agent. Studies of other closely related *Bacillus* and *Clostridium* spp. suggest that the surface of the endospore is covered with a hair‐like nap which is made up of collagen‐like fibres and there is growing evidence now that *Pasteuria* endospores also have a similar fibrous hair‐like nap made up of glycosylated CLPs (Davies & Opperman, 2006; Orr et al., 2018) which we show here to play an important role in endospore attachment. Bioinformatics has shown that in *Pasteuria* CLPs are a diverse group of sequences possibly obtained through horizontal gene transfer (Srivastava et al., 2018). If the diverse range of collagen‐like sequences in *P. penetrans* have been obtained from outside that clade, this begs the question as to the nature of these adhesive qualities and methods by which they can be studied. To this end, it was decided to explore the similarities between *P. penetrans* and *B. thuringiensis* endospores the latter being easy to mass produce and, therefore, the possibility that it could be developed into a gain of function model.

Silver staining of the endospore extracts suggests some considerable similarities in the endospore protein composition between *P. penetrans* and three different strains of *B. thuringiensis*. More bands were revealed in the endospore extracts from the three *B. thuringiensis* strains than in *P. penetrans*. As evident from the total protein profile, there was at least one protein (~58 kDa) that was commonly found in *P. penetrans* and all the three *B. thuringiensis* strains. In an earlier study a 58 kDa protein, not revealed by silver staining, appeared on the Western blot of endospore extracts of three different populations of *P. penetrans* when probed with a polyclonal antibody raised to *P. penetrans* (Davies et al., 1992). Strikingly, the molecular chaperone protein GroEL, which is known to be abundant in bacterial exosporium, also migrates with an apparent molecular weight of 58 kDa (Charlton et al., 1999; Redmond et al., 2004); GroEL is present throughout the tree of life, although currently its function in the endospore remains unknown. Another protein common to both *P. penetrans* and *B. thuringiensis* Kurstaki cry‐had a molecular weight of ~19 kDa, but this peptide was not observed in the other two *B. thuringiensis* strains. Interestingly, a 17 kDa protein ExsF/BxpB known to be associated with the exosporium of *Bacillus* species (Redmond et al., 2004) was similar to a 17 kDa polypeptide observed in *P. penetrans* (Davies et al., 1992). Two bands of ~112 and ~42 kDa, respectively, were unique to *P. penetrans* endospores.

The Western blot analyses showed the presence of several endospore proteins with common epitopes between *P. penetrans* and *B. thuringiensis*. The *Pasteuria*‐specific polyclonal antibody Anti‐PpWS recognized a >250 kDa protein in *B. thuringiensis* strains but no such high molecular weight band in *P. penetrans*. A similar >250 kDa protein band was seen in all the bacteria, including *P. penetrans*, when the blot was probed with Col1981 and in *P. penetrans* when probed with Col1982. Both Col1981 and Col1982 are antibodies raised to two different collagen‐like synthetic polypeptides originated from *Pasteuria* GSS database. The above results suggest a commonality in the collagen‐like endospore proteins between *P. penetrans* and *B. thuringiensis*. The BclA protein in the exosporium of *Bacillus* spp. is known to migrate with an apparent molecular weight of >250 kDa due to heavy glycosylation (Sylvestre et al., 2002), and interestingly, our glycoprotein staining revealed bands of >250 kDa in both *P. penetrans* and *B. thuringiensis* Al Hakam and Kusrtaki cry‐strains.

Another protein band of ~72 kDa band was also commonly recognized by the Anti‐PpWS antibody in all the protein samples. Clearly, Anti‐PpWS detected a common epitope in these endospore proteins that have a comparable molecular weight in *P. penetrans* and the three *B. thuringiensis* strains. Col1982 antibody detected a similar band in *P. penetrans*. This suggests that the ~72 kDa band in *P. penetrans* is a CLP. These ~72 kDa bands recognized by Anti‐PpWS in (*P. penetrans* and *B. thuringiensis*) may represent orthologous proteins that differ in a specific epitope that is recognized by Col1982 in *P. penetrans*, suggesting it is either not present in the *B. thuringiensis* orthologue or is not conformationally accessible for the binding of Col1982.

Staining with WGA revealed an ~139 kDa protein band in *B. thuringiensis* Al Hakam, previously recognized by Col1981, suggesting it is a collagen‐like glycoprotein decorated with NAG sugars, but no other bands were observed in either of the other two strains of *B. thuringiensis*. WGA showed another six bands (>250, ~224, ~176, ~126, ~83 and ~72 kDa) in *P. penetrans* of which the ~126 and ~83 kDa protein bands are comparable in size and similar to two bands previously reported in *P. penetrans* endospores, and both bearing terminal NAG residues (Persidis et al., 1991). The current study conveys that *P. penetrans* contains at least six endospore proteins that are glycosylated with NAG, two of which are collagen‐like glycoproteins detected by Col1982.

Although early experiments had no of little effect, collagenase treatment of 6 h prior to gel electrophoresis and Western blot analysis reduced the number of bands detected by WGA from six to only three bands, suggesting that those which disappeared or moved position were collagenous. These results indicate the presence of CLPs in the endospores of *P. penetrans* and *B. thuringiensis* and that some of them are glycosylated with NAG. Although others have studied the regulation and structure of CLPs in *B. thuringiensis* endospores (Peng et al., 2016; Terry et al., 2017) their functional role in the life cycle of *B. thuringiensis* is a matter for conjecture. The fact that CLPs of *P. penetrans* endospores are almost certainly involved in attachment to the nematode cuticle suggests that they may play a similar functional role in *B. thuringiensis*. Building on the Western blot analysis above our immunolocalization studies suggest common collagen‐like epitopes on the endospore surface of *P. penetrans* and *B. thuringiensis* showing that Anti‐PpWS recognized surface localized epitopes of both *Pasteuria* and *Bacillus* endospores and that the endospore surfaces were also recognized by both the *Pasteuria‐*collagen specific antibodies, Col1981 and Col1982.

Our observation that surface‐localized epitopes of both *Pasteuria* and *Bacillus* endospores were recognized by the antibody raised to whole endospore of *P. penetrans* (Anti‐PpWS) and a *Pasteuria‐*collagen specific antibody (Col1982) suggests that one or more endospore proteins (including one or more CLPs), characterized in our immunoblotting experiments, were surface associated. As these antibodies were either raised to *P. penetrans* endospores or to synthetic CL peptides of *P. penetrans*, the observed differences in the fluorescence intensity of *B. thuringiensis* and *Pasteuria* endospores were obvious. Interestingly the central body of the *Pasteuria* endospores probed with Anti‐ppWS was not recognized revealing two concentric circles, one just outlining the central body, and the other recognizing the outer surface of the exosporium. All *B. thuringiensis* endospores were also recognized by Anti‐PpWS, prominently on the periphery indicating shared epitopes between *P. penetrans* and *B. thuringiensis* exsoporium. When probed with the *Pasteuria‐*specific CL‐peptide antibody, Col1982, *Pasteuria* and *Bacillus* endospores showed similar fluorescence patterns suggesting that some common collagen‐like epitopes are present on the exosporium of the endospores in both *Pasteuria* and *B. thuringiensis*.

Previously, an immuno‐localization based study using a monoclonal antibody raised to whole endospores of *Pasteuria* indicated that an endospore‐associated surface epitope was almost uniformly localized on the periphery of *Pasteuria* endospores (Brito et al., 2003). Several other studies using other monoclonal and polyclonal antibodies suggested surface epitope heterogeneity among individual endospores of *P. penetrans* within and between populations; this heterogeneity has been linked to their host preferences and specificities (Davies et al., 1994; Davies & Redden, 1997). However, this is the first study where collagen‐specific antibodies have been used to characterize *Pasteuria* endospore surface. Formerly, the localization of the BclA protein on the surface of *Bacillus* endospores has been studied using immuno‐labelling with anti‐BclA antibodies (Sylvestre et al., 2002; Thompson & Stewart, 2008). BclA is a CLP localized in the exosporium of *Bacillus* spp. that has been shown to be involved in host interaction (Bozue et al., 2007; Gu et al., 2012). Since Col1981 and Col1982 recognized epitopes on the exosporium of *B. thuringiensis*, it can be speculated that the epitope recognized by the two antibodies could possibly be a part of a BclA‐like protein. If this is true, it is expected that this CLP in *Pasteuria* could play major role in nematode attachment.

Probing *Pasteuria* endospores with FITC‐labelled WGA indicated the presence of NAG in a pattern similar to that exhibited by Anti‐PpWS, that is, fluorescence decorated in two concentric circles. Again, the central body of the endospores were not recognized indicating that the signal was coming from the exosporia of *Pasteuria* and *Bacillus* endospores, most likely the parasporal fibres. When the endospores pre‐treated with WGA were probed with Anti‐PpWS, there was a marked decrease in the observed recognition suggesting that WGA was binding to NAG residues and masking the sugar moieties on the endospore surface. However, steric hindrance caused by the large WGA molecule (WGA) cannot be ruled out. Unexpectedly, following endospore treatment with WGA the fluorescence increased when they were probed with Col1982 antibody suggesting that Col1982 was less specific or more cross‐reactive. Previous studies using lectins have shown the endospore surface‐associated proteins of *Pasteuria* to be linked to NAG and NAM residues, and suggest that these two sugar residues form a key functional role in the interaction between endospores and the second‐stage juvenile cuticle (Bird et al., 1989; Davies & Danks, 1993; Davies & Redden, 1997; Persidis et al., 1991; Spiegel et al., 1996).

Work in the early 1990s suggested that *Pasteuria* endospores bound to nematode cuticle through a combination of protein and carbohydrate interactions (Davies & Danks, 1993; Persidis et al., 1991; Spiegel et al., 1996); however, the nature of these interactions and the mechanism of host specificity was poorly understood. The work reported here suggests that collagen‐like fibres on the surface of the endospore are decorated with NAG and are the basis for attachment.

A striking observation of our attachment assays was the high frequency of unconventionally attached endospores when either endospore or J2 was pre‐treated with collagenase or NAGase or WGA. A former study reported inverted attachment when endospores of an isolate of *Pasteuria* from pigeon pea cyst nematodes, *Heterodera cajani*, were found to cross‐infect potato cyst nematodes, *Globodera pallida* (Mohan et al., 2012). It was then suggested that the inverted attachment was possibly due to a difference in the molecular receptors of the two genera of nematodes. The current attachment assays were done on a single population of a single genus of nematodes; therefore, the above explanation is not applicable here. The conventional orientation of endospores, where the concave surface of the skirt‐like structure formed by parasporal fibres orients itself towards the nematode cuticle, has been previously explained by the presence of denser collagen‐like fibres on the concave side than on the convex side (Davies, 2009). Notwithstanding the fact that the endospore attachment studies were undertaken in India and on root‐knot nematodes cultured on cowpea, and therefore may have exhibited slightly different attachment specificites, it is interesting to note that, in this study, all control treatments (endospores and J2s treated in PBS), involving same set of endospores and J2s as used for various treatments, exhibited conventional attachments. Thus, it is apparent that unconventional attachment is triggered by collagenase, NAGase or WGA treatments.

We know that the *P. penetrans* genome contains a diverse set of genes that encode CLPs (Orr et al., 2018; Srivastava et al., 2018) and it is possible that different sets of collagen‐like adhesins are expressed on the different surfaces of the endospores. It is also likely that the treatments of endospores do not affect the convex and concave surfaces of the endospores equally, and hence produce changes in binding orientation. The attachment of *Pasteuria* endospores to the nematode cuticle appears to be a complex molecular process which involves both CLPs and sugar moieties, NAG, present on the *Pasteuria* endospore surface. The observation that the surface of endospores of *B. thuringiensis* also have similar glycosylated collagens suggests they may make a good “gain of function” bacterial platform which can be used to experimentally manipulate endospore adhesion and aid our understanding of host specificity between *Pasteuria* and plant‐parasitic nematodes.

## CONFLICT OF INTEREST

K.G.D. is Director of KG Davies Ltd a small crop protection consultancy.

## Supporting information


Figure S1

Data S1

Data S2
