# Supplementary figures and images for: Exploring Bacillus thuringiensis as a model for endospore adhesion and its potential to investigate adhesins in Pasteuria penetrans
Source: J Appl Microbiol. 2022 Mar 22;132(6):4371–87. doi: 10.1111/jam.15522 (PMC9311801; doi:10.1111/jam.15522)

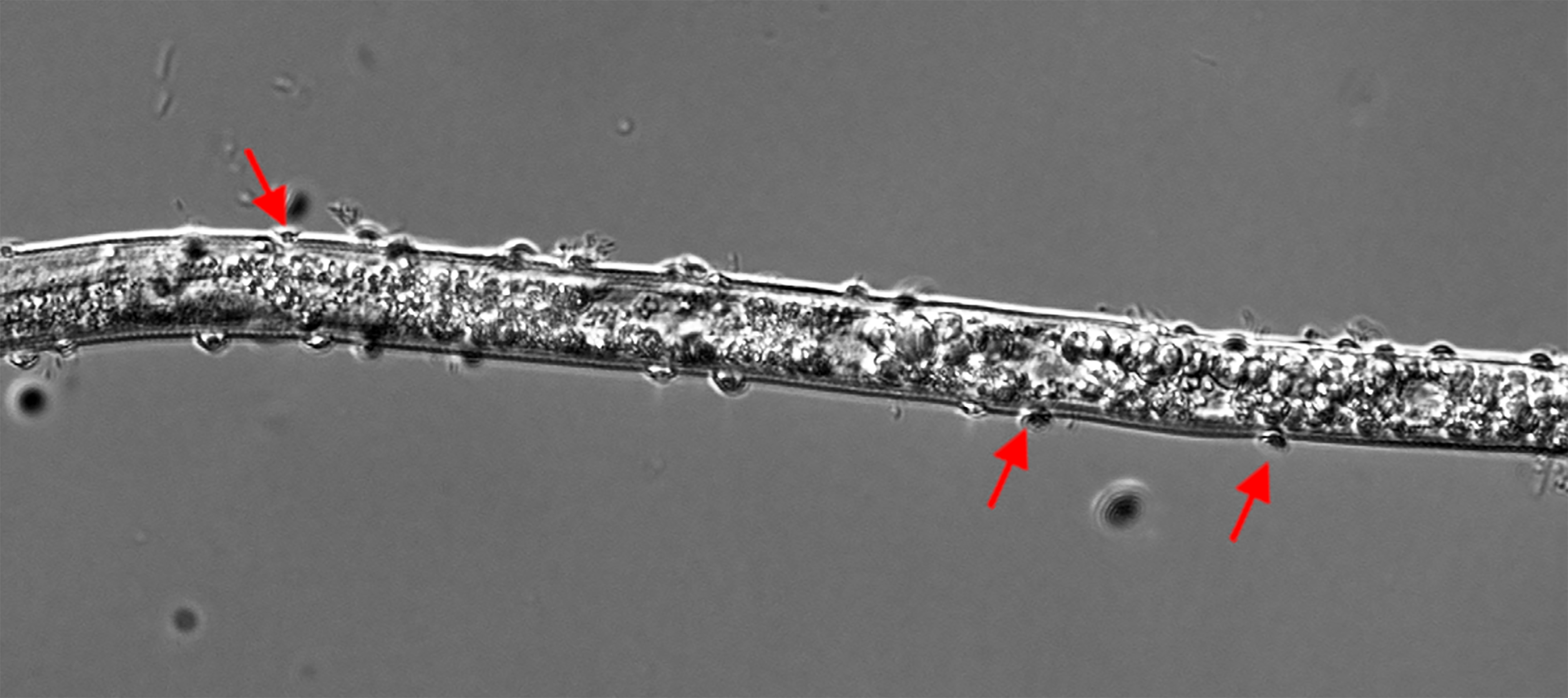

Supplement: Supplementary file 1 — Figure S1 [file JAM-132-4371-s001.tif]
