# Supplementary material for: Exploring Bacillus thuringiensis as a model for endospore adhesion and its potential to investigate adhesins in Pasteuria penetrans
Source: J Appl Microbiol. 2022 Mar 22;132(6):4371–87. doi: 10.1111/jam.15522 (PMC9311801; doi:10.1111/jam.15522)
Supplement: Supplementary file 2 — Data S1 [file JAM-132-4371-s003.docx]

**SUPPLEMENTARY MATERIAL I (MEDIA)**

1. **Composition of Sporulation broth**

Nutrient broth (Oxoid) 8 g

KCl (10% w/v) 10 ml

MgSO_4_.7H_2_O (1.2%) 10 ml

NaOH (1M) 1.5 ml

Ca(NO_3_)_2_.4H_2_O (1M) 1 ml

MnCl_2_ (0.01M) 1 ml

FeSO_4_.7H_2_O (1 mM) 1 ml

Steps to prepare:

1. Nutrient broth, KCl, MgSO_4_.7H_2_O, NaOH, Ca(NO_3_)_2_.4H_2_O, MnCl_2_, FeSO_4_.7H_2_O were dissolved in 1 litre d.w. and the pH was adjusted to 7.6.
2. The above solution was sterilized in an autoclave at 121°C for 15 minutes.
3. Ca(NO_3_)_2_.4H_2_O, MnCl_2_ (0.01M) and FeSO_4_.7H_2_O (1 mM) solutions were made separately in de-ionized water and filter sterilized.
4. The autoclaved and filter sterilized portions of the media were mixed.
5. **Schaeffer-Fulton technique of endospore staining**

Bacillus endospores were stained using malachite green and safranin by the differential endospore staining technique as described by (Schaeffer and Fulton, 1933). This technique exploits the fact that the endospore wall is thick and resistant and does not allow a stain to permeate easily. Malachite green is used as a primary stain to stain endospores which enters the spore wall while the endospores are being gently heated on a slide. Once the heating is stopped, the green coloured stain is locked inside the spore wall integuments. At this stage, malachite green stain enters the vegetative cells as well but is easily washed away with water. Counterstaining with safranin stains vegetative cells in red. For the staining, *Bacillus* cells were smeared on clean glass slides, heat-fixed, and the slides were kept over a steaming water bath on a stand. The smears were drenched in malachite green while there was continuous steaming from underneath. This was done for 10 minutes and the stain was not allowed to dry. The green-stained smear was washed with tap water and counterstained with safranin for a minute. The slides were washed thoroughly with tap water till the running off water becomes clear. The slides were observed under 100X objective of a light microscope (Leitzlaborlux, Leica Mikroskope und Systeme GmbH, Wetzlar, Germany) using immersion oil.

1. **Reagents for SDS-PAGE**

**Tris/HCl, 1.5 M pH 8.8**

Tris base 18.15 g

De-ionized water 100 ml

pH adjusted to 8.8 with HCl

*Stored at 4°C*

**Tris/HCl, 0.5 M pH 6.8**

Tris base 6 g

De-ionized water 100 ml

pH adjusted to 6.8 with HCl

*Stored at 4°C*

**Acrylamide Stock, 30%**

Readymade

*Stored at 4°C*

**TEMED**

Readymade

*Stored at 4°C*

**10%APS (Ammonium persulphate) in de-ionized water**

*Made fresh*

**SDS (Sodium dodecyl suphate) 10%**

*Stored at RT*

**Electrode Buffer (2X)**

Tris 6.06 g

Glycine 28.8 g

SDS 2 g

De-ionized water 1 litre

Steps to prepare:

1. Tris and Glycine were dissolved in ~800 ml d.w.
2. SDS was added to the above.
3. The volume was made up to 1 litre.

**Sample Buffer**

Tris/HCl, 0.5 M pH 6.8 2.5 ml

Glycerol 2 ml

SDS (10%) 4 ml

Bromophenol blue (1%) 0.4 ml

DTT 0.1 ml

De-ionized water 1 ml

Total 10 ml

*The sample buffer was made without DTT and stored at RT.*

*DTT from stock was added freshly before use.*

**Resolving Gel (For 2 gels)**

7**% 12%**

Acrylamide stock 30% 2.33 ml 4 ml

1.5 M Tris/HCl, pH 8.8 1.75 ml 2.5 ml

De-ionized water 5.765 ml 3.345 ml

SDS (10%) 100 µl 100 µl

APS (10%) 50 µl 50 µl

TEMED 5 µl 5 µl

**Stacking Gel (For 2 gels)**

**4%**

Acrylamide stock 30% 1.33 ml

1.5 M Tris/HCl, pH 8.8 1.25 ml

De-ionized water 2.34 ml

SDS (10%) 50 µl

APS (10%) 25 µl

TEMED 5 µl

1. **Reagents for Western Blot and colourimetric detection**

**Blotting Buffer**

Tris 6.06 g

Glycine 28.83 g

Water 1600 ml

Methanol ~400 ml

Steps to prepare:

- - 1. Tris and Glycine were dissolved in water.
    2. Methanol was added.
    3. Volume was made up to 2 litres.

*Stored at 4°C*

**Methanol**

*To soak PVDF membranes before blotting*

*Stored at 4°C*

**Washing Solution (PBS-Tween 1X)**

Sodium Chloride 8.0 g

Potassium Chloride 0.2 g

Disodium hydrogen orthophosphate (anhydrous) 1.44 g

Potassium dihydrogen orthophosphate 0.24 g

Tween 80* 0.5 ml

Steps to prepare:

1. The above ingredients (except tween 80) were dissolved in ~800 ml de-ionized water.
2. The pH of the solution was adjusted to 7.4.
3. The volume was made up to 1 litre.
4. Tween 80 added gradually to rapidly stirred solution

*Stored at 4°C*

**Blocking Solution (2% Tween 80 in PBS)**

Washing Solution (1X) 100 ml

Tween 80 1.95 ml

Skimmed Milk Powder* 1%

*Stored at 4°C*

**Skimmed milk powder to be added just before use.*

**Substrate for colourimetric detection**

BCIP* 2 mg/ml in DEA buffer, 10 mg needed per blot

**Made fresh*

10% DEA buffer

Diethanolamine 97 ml

Magnesium chloride hexahydrate 100 mg

Steps to prepare the DEA buffer:

1. The above two ingredients were mixed in 800 ml d.w.
2. The pH was adjusted to 9.8 with HCl.
3. The volume was made up to 1 litre.

*DEA buffer was stored at 4°C.*

1. **VECTASTAIN^®^ABC-AP Reagent (Vector Laboratories, UK)**

Two drops of Reagent A (Avidin DH solution) was mixed with two drops of Reagent B (biotinylated alkaline phosphatase) in the provided reagent mixing bottle and allowed to stand for 30 minutes at room temperature before use.
