# Supplementary material for: Exploring Bacillus thuringiensis as a model for endospore adhesion and its potential to investigate adhesins in Pasteuria penetrans
Source: J Appl Microbiol. 2022 Mar 22;132(6):4371–87. doi: 10.1111/jam.15522 (PMC9311801; doi:10.1111/jam.15522)
Supplement: Supplementary file 3 — Data S2 [file JAM-132-4371-s002.docx]

**SUPPLEMENTARY MATERIAL (Molecular Weights)**

1. **Estimation of molecular weight of proteins from SDS-PAGE gels and Western blots**

To determine the molecular weight of protein/polypeptide components in the endospore protein extracts, after SDS-PAGE/ Western blotting, the images of the stained gels/blots were digitally captured and analyzed using the image processing and analysis software tool Fiji (Schindelin et al., 2012). The migration distances (Y) of each visible band of the protein samples and the molecular markers were measured from the top of each resolving gel. The relative migration distance (R_f_) of each band of the molecular marker and the endospore proteins, was determined using the following equation:

$$\boldsymbol{R}_{\boldsymbol{f}}\boldsymbol{=}\frac{\boldsymbol{Migration distance of protein}}{\boldsymbol{Length of the resolving gel}}$$

Graphs were plotted using the values of R_f_ obtained for the molecular markers on the x-axis and the logarithmic values of the known molecular weights of the molecular markers on the y-axis. Lines of best fit were drawn and the consequent straight-line equations were derived. The molecular weights of the unknown proteins were determined by interpolating their R_f_ values into the straight-line equation. The following tables show the distance of migration ‘Y’ of each band (as measured from the images of the gels and plots), the relative distance of migration (R_f_), the molecular weight of each molecular marker, and all the calculations done to determine the molecular weight of the unknown proteins.

**Table 1: Silver Stained Gel (Total protein profile)**

| S.No. | Lane | Band | Y | Rf | M.W (kDa) | log MW | MW of unknown |
| --- | --- | --- | --- | --- | --- | --- | --- |
| 1 | Molecular marker | Band 1 | 229.3 | 0.293974 | 200 | 2.30103 | -- |
| 2 | Molecular marker | Band 2 | 268 | 0.34359 | 116 | 2.064458 | -- |
| 3 | Molecular marker | Band 3 | 289.3 | 0.370897 | 97 | 1.986772 | -- |
| 4 | Molecular marker | Band 4 | 365.3 | 0.468333 | 66 | 1.819544 | -- |
| 5 | Molecular marker | Band 5 | 433.3 | 0.555513 | 45 | 1.653213 | -- |
| 6 | Molecular marker | Band 6 | 545.3 | 0.699103 | 31 | 1.491362 | -- |
| 7 | Molecular marker | Band 7 | 682.7 | 0.875256 | 21 | 1.322219 | -- |
| 8 | Molecular marker | Band 8 | 756 | 0.969231 | 6 | 0.778151 | -- |
| 9 | **BT-Al Hakam** | **Band 2** | 234.7 | 0.300897 | ? | 2.178759 | **150.9243** |
| 10 | **BT-Al Hakam** | **Band 3** | 249.3 | 0.319615 | ? | 2.14412 | **139.3541** |
| 11 | **BT-Al Hakam** | **Band 6** | 298.7 | 0.382949 | ? | 2.026915 | **106.3935** |
| 12 | **BT-Al Hakam** | **Band 7** | 333.3 | 0.427308 | ? | 1.944824 | **88.06927** |
| 13 | **BT-Al Hakam** | **Band 8** | 354.7 | 0.454744 | ? | 1.894052 | **78.35226** |
| 14 | **BT-Al Hakam** | **Band 9** | 374.7 | 0.480385 | ? | 1.8466 | **70.24254** |
| 15 | **BT-Al Hakam** | **Band 10** | 406.7 | 0.52141 | ? | 1.770678 | **58.97639** |
| 16 | **BT-Al Hakam** | **Band 12** | 502.7 | 0.644487 | ? | 1.542912 | **34.90696** |
| 17 | **BT-Al Hakam** | **Band 13** | 522.7 | 0.670128 | ? | 1.495461 | **31.29398** |
| 18 | **BT-Al Hakam** | **Band 14** | 549.3 | 0.704231 | ? | 1.432351 | **27.06142** |
| 19 | **BT-Al Hakam** | **Band 15** | 573.3 | 0.735 | ? | 1.375409 | **23.73608** |
| 20 | **BT-Al Hakam** | **Band 17** | 629.3 | 0.806795 | ? | 1.242545 | **17.48016** |
| 21 | **BT-Al Hakam** | **Band 18** | 657.3 | 0.842692 | ? | 1.176114 | **15.00077** |
| 22 | **BT-Al Hakam** | **Band 21** | 690.7 | 0.885513 | ? | 1.09687 | **12.49885** |
| 23 | **BT-Al Hakam** | **Band 22** | 712 | 0.912821 | ? | 1.046334 | **11.12588** |
| 24 | **BT-Al Hakam** | **Band 24** | 757.3 | 0.970897 | ? | 0.938857 | **8.686748** |
| 25 | **BT-Al Hakam** | **Band 25** | 773.3 | 0.99141 | ? | 0.900896 | **7.95969** |
| 26 | **BT-kurstaki cry-** | **Band 1** | 221.3 | 0.283718 | ? | 2.210552 | **162.3871** |
| 27 | **BT-kurstaki cry-** | **Band 2** | 233.3 | 0.299103 | ? | 2.182081 | **152.083** |
| 28 | **BT-kurstaki cry-** | **Band 5** | 297.3 | 0.381154 | ? | 2.030237 | **107.2103** |
| 29 | **BT-kurstaki cry-** | **Band 10** | 414.7 | 0.531667 | ? | 1.751698 | **56.45438** |
| 30 | **BT-kurstaki cry-** | **Band 12** | 505.3 | 0.647821 | ? | 1.536743 | **34.41465** |
| 31 | **BT-kurstaki cry-** | **Band 13** | 532 | 0.682051 | ? | 1.473396 | **29.74376** |
| 32 | **BT-kurstaki cry-** | **Band 15** | 566.7 | 0.726538 | ? | 1.391068 | **24.60752** |
| 33 | **BT-kurstaki cry-** | **Band 16** | 613.3 | 0.786282 | ? | 1.280506 | **19.07684** |
| 34 | **BT-kurstaki cry-** | **Band 17** | 636 | 0.815385 | ? | 1.226649 | **16.85191** |
| 35 | **BT-kurstaki cry-** | **Band 19** | 668 | 0.85641 | ? | 1.150727 | **14.14905** |
| 36 | **BT-kurstaki cry-** | **Band 21** | 693.3 | 0.888846 | ? | 1.090701 | **12.32257** |
| 37 | **BT-kurstaki cry-** | **Band 25** | 774.7 | 0.993205 | ? | 0.897575 | **7.899045** |
| 38 | **BT-berliner** | **Band 1** | 220 | 0.282051 | ? | 2.213636 | **163.5445** |
| 39 | **BT-berliner** | **Band 2** | 234.7 | 0.300897 | ? | 2.178759 | **150.9243** |
| 40 | **BT-berliner** | **Band 9** | 374.7 | 0.480385 | ? | 1.8466 | **70.24254** |
| 41 | **BT-berliner** | **Band 10** | 409.3 | 0.524744 | ? | 1.76451 | **58.14462** |
| 42 | **BT-berliner** | **Band 12** | 505.3 | 0.647821 | ? | 1.536743 | **34.41465** |
| 43 | **BT-berliner** | **Band 13** | 530.7 | 0.680385 | ? | 1.47648 | **29.95575** |
| 44 | **BT-berliner** | **Band 15** | 566.7 | 0.726538 | ? | 1.391068 | **24.60752** |
| 45 | **BT-berliner** | **Band 17** | 630.7 | 0.80859 | ? | 1.239224 | **17.34698** |
| 46 | **BT-berliner** | **Band 18** | 656 | 0.841026 | ? | 1.179198 | **15.10769** |
| 47 | **BT-berliner** | **Band 20** | 676 | 0.866667 | ? | 1.131747 | **13.54399** |
| 48 | **BT-berliner** | **Band 23**  **(start of smear)** | 686.7 | 0.880385 | ? | 1.10636 | **12.77498** |
| 49 | **BT-berliner** | **Band 23**  **(end of smear)** | 758.7 | 0.972692 | ? | 0.935536 | **8.620563** |
| 50 | **BT-berliner** | **Band 25** | 770.7 | 0.988077 | ? | 0.907065 | **8.073556** |
| 51 | **Pasteuria** | **Band 4** | 289.3 | 0.370897 | ? | 2.049217 | **111.9998** |
| 52 | **Pasteuria** | **Band 10** | 405.3 | 0.519615 | ? | 1.774 | **59.42918** |
| 53 | **Pasteuria** | **Band 11** | 469.3 | 0.601667 | ? | 1.622156 | **41.89437** |
| 54 | **Pasteuria** | **Band 16** | 609.3 | 0.781154 | ? | 1.289997 | **19.4983** |

*Length of the resolving gel = 780

**Table 2: Immunodetection with AntiPpWS (Western Blot)**

| S.No. | Lane | Band | Y | Rf* | M.W (kDa) | log MW | MW of unknown |
| --- | --- | --- | --- | --- | --- | --- | --- |
| 1 | Molecular marker | Band 1 | 232 | 0.405594 | 250 | 2.39794 | -- |
| 2 | Molecular marker | Band 2 | 309 | 0.54021 | 150 | 2.176091 | -- |
| 3 | Molecular marker | Band 3 | 412 | 0.72028 | 100 | 2 | -- |
| 4 | Molecular marker | Band 4 | 495 | 0.865385 | 75 | 1.875061 | -- |
| 5 | **BT-Al Hakam** | **Band 1** | 228 | 0.398601 | ? | 2.373178 | **236.1444** |
| 6 | **BT-Al Hakam** | **Band 3** | 492 | 0.86014 | ? | 1.859162 | **72.30399** |
| 7 | **BT-kurstaki cry-** | **Band 1** | 227 | 0.396853 | ? | 2.375125 | **237.2054** |
| 8 | **BT-kurstaki cry-** | **Band 2** | 422 | 0.737762 | ? | 1.995454 | **98.95875** |
| 9 | **BT-kurstaki cry-** | **Band 4** | 498 | 0.870629 | ? | 1.84748 | **70.38499** |
| 10 | **BT-berliner** | **Band 1** | 221 | 0.386364 | ? | 2.386807 | **243.6727** |
| 11 | **BT-berliner** | **Band 2** | 413 | 0.722028 | ? | 2.012977 | **103.0333** |
| 12 | **BT-berliner** | **Band 4** | 498 | 0.870629 | ? | 1.84748 | **70.38499** |
| 13 | **Pasteuria** | **Band 4** | 493 | 0.861888 | ? | 1.857215 | **71.98056** |

*Length of the resolving gel = 572

**Table 3: Immunodetection with Col1981 (Western Blot)**

| S.No. | Lane | Band | Y | Rf* | M.W (kDa) | log MW | MW of unknown |
| --- | --- | --- | --- | --- | --- | --- | --- |
| 1 | Molecular marker | Band 1 | 366.7 | 0.485888 | 250 | 2.39794 | -- |
| 2 | Molecular marker | Band 2 | 482.7 | 0.639592 | 150 | 2.176091 | -- |
| 3 | Molecular marker | Band 3 | 624.8 | 0.827879 | 100 | 2 | -- |
| 4 | Molecular marker | Band 4 | 731.3 | 0.968994 | 75 | 1.875061 | -- |
| 5 | **BT-Al Hakam** | **Band 2** | 340.7 | 0.451438 | ? | 2.409564 | **256.7817** |
| 6 | **BT-Al Hakam** | **Band 4** | 530.1 | 0.702398 | ? | 2.142316 | **138.7765** |
| 7 | **BT-kurstaki cry-** | **Band 1** | 329 | 0.435935 | ? | 2.426073 | **266.7307** |
| 8 | **BT-kurstaki cry-** | **Band 3** | 496 | 0.657215 | ? | 2.190432 | **155.0358** |
| 9 | **BT-berliner** | **Band 1** | 329 | 0.435935 | ? | 2.426073 | **266.7307** |
| 10 | **BT-berliner** | **Band 5** | 620.9 | 0.822711 | ? | 2.014195 | **103.3225** |
| 11 | **BT-berliner** | **Band 6** | 651.2 | 0.862859 | ? | 1.971441 | **93.6356** |
| 12 | **Pasteuria** | **Band 1** | 330 | 0.43726 | ? | 2.424662 | **265.8655** |

*Length of the resolving gel= 754.7

**Table 4: Immunodetection with Col1982 (Western Blot)**

| S.No. | Lane | Band | Y | Rf | M.W (kDa) | log MW | MW of unknown |
| --- | --- | --- | --- | --- | --- | --- | --- |
| 1 | Molecular marker | Band 1 | 361.2 | 0.478601 | 250 | 2.39794 | -- |
| 2 | Molecular marker | Band 2 | 479.4 | 0.635219 | 150 | 2.176091 | -- |
| 3 | Molecular marker | Band 3 | 620 | 0.821518 | 100 | 2 | -- |
| 4 | Molecular marker | Band 4 | 735.2 | 0.974162 | 75 | 1.875061 | -- |
| 5 | **BT-Al Hakam** | **Band 2** | 490 | 0.649265 | ? | 2.193636 | **156.184** |
| 6 | **BT-berliner** | **Band 4** | 597.5 | 0.791705 | ? | 2.045185 | **110.9647** |
| 7 | **BT-berliner** | **Band 5** | 620.9 | 0.822711 | ? | 2.012871 | **103.0079** |
| 8 | **Pasteuria** | **Band 1** | 353.4 | 0.468266 | ? | 2.382274 | **241.1424** |
| 9 | **Pasteuria** | **Band 3** | 519.4 | 0.68822 | ? | 2.153037 | **142.2449** |
| 10 | **Pasteuria** | **Band 6** | 661 | 0.875845 | ? | 1.957495 | **90.67648** |
| 11 | **Pasteuria** | **Band 7** | 734.2 | 0.972837 | ? | 1.856409 | **71.84713** |

*Length of the resolving gel= 745.7

**Table 5: Glycoprotein staining of gels**

| S.No. | Lane | Band | Y | Rf | M.W (kDa) | log MW | MW of unknown |
| --- | --- | --- | --- | --- | --- | --- | --- |
| 1 | Molecular marker | Band 1 | 223 | 0.337368 | 250 | 2.39794 | -- |
| 2 | Molecular marker | Band 2 | 255 | 0.385779 | 150 | 2.176091 | -- |
| 3 | Molecular marker | Band 3 | 301 | 0.455371 | 100 | 2 | -- |
| 4 | Molecular marker | Band 4 | 340 | 0.514372 | 75 | 1.875061 | -- |
| 5 | Molecular marker | Band 5 | 419 | 0.633888 | 50 | 1.69897 | -- |
| 6 | Molecular marker | Band 6 | 496 | 0.750378 | 37 | 1.568202 | -- |
| 7 | Molecular marker | Band 7 | 607 | 0.918306 | 25 | 1.39794 | -- |
| 8 | Molecular marker | Band 8 | 654 | 0.98941 | 20 | 1.30103 | -- |
| 9 | **BT-Al Hakam** | **Band 2** | 211 | 0.319213 | ? | 2.265504 | **184.2909** |
| 10 | **BT-kurstaki cry-** | **Band 2** | 213 | 0.322239 | ? | 2.260889 | **182.3429** |
| 11 | **BT-kurstaki cry-** | **Band 3**  **(start of smear)** | 405 | 0.612708 | ? | 1.817836 | **65.74102** |
| 12 | **BT-kurstaki cry-** | **Band 3**  **(end of smear)** | 486 | 0.73525 | ? | 1.630924 | **42.74878** |
| 13 | **Pasteuria** | **Band 1** | 203 | 0.30711 | ? | 2.283964 | **192.2934** |

*Length of the resolving gel = 661

**Table 6:** **Detection of glycoproteins by Lectin blotting (Western Blot)**

| S.No. | Lane | Band | Y | Rf | M.W (kDa) | log MW | MW of unknown |
| --- | --- | --- | --- | --- | --- | --- | --- |
| 1 | Molecular marker | Band 1 | 322 | 0.438692 | 250 | 2.39794 | -- |
| 2 | Molecular marker | Band 2 | 440 | 0.599455 | 150 | 2.176091 | -- |
| 3 | Molecular marker | Band 3 | 577 | 0.786104 | 100 | 2 | -- |
| 4 | Molecular marker | Band 4 | 703 | 0.957766 | 75 | 1.875061 | -- |
| 5 | **BT-Al Hakam** | **Band 4** | 488 | 0.66485 | ? | 2.142813 | **138.9355** |
| 6 | **Pasteuria** | **Band 1** | 247 | 0.336512 | ? | 2.470527 | **295.4793** |
| 7 | **Pasteuria** | **Band 2** | 335 | 0.456403 | ? | 2.350864 | **224.3179** |
| 8 | **Pasteuria** | **Band 3** | 412 | 0.561308 | ? | 2.246159 | **176.262** |
| 9 | **Pasteuria** | **Band 5** | 520 | 0.708447 | ? | 2.099299 | **125.6896** |
| 10 | **Pasteuria** | **Band 6** | 654 | 0.891008 | ? | 1.917085 | **82.61991** |
| 11 | **Pasteuria** | **Band 7** | 713 | 0.97139 | ? | 1.836856 | **68.68407** |

*Length of the resolving gel = 734

**Table 7: Detection of collagens with NAG as glyco-conjugate after 6 h collagenase treatment**

| S.No. | Lane | Band | Y | Rf | M.W (kDa) | log MW | MW of unknown |
| --- | --- | --- | --- | --- | --- | --- | --- |
| 1 | Molecular marker | Band 1 | 360.1 | 0.547264 | 250 | 2.39794 | -- |
| 2 | Molecular marker | Band 2 | 419.9 | 0.638146 | 150 | 2.176091 | -- |
| 3 | Molecular marker | Band 3 | 521.8 | 0.793009 | 100 | 2 | -- |
| 4 | Molecular marker | Band 4 | 613.1 | 0.931763 | 75 | 1.875061 | -- |
| 5 | **Pasteuria (untreated)** | **Band 1** | 325.5 | 0.494681 | ? | 2.41655 | **260.9456** |
| 6 | **Pasteuria (untreated)** | **Band 2** | 384.2 | 0.583891 | ? | 2.299989 | **199.521** |
| 7 | **Pasteuria (untreated)** | **Band 3** | 451.4 | 0.686018 | ? | 2.166549 | **146.74** |
| 8 | **Pasteuria (untreated)** | **Band 6** | 559.6 | 0.850456 | ? | 1.951694 | **89.47347** |
| 9 | **Pasteuria (untreated)** | **Band 8** | 616.3 | 0.936626 | ? | 1.839104 | **69.04056** |
| 10 | **Pasteuria (treated)** | **Band 4** | 475.6 | 0.722796 | ? | 2.118494 | **131.3694** |
| 11 | **Pasteuria (treated)** | **Band 5** | 505 | 0.767477 | ? | 2.060114 | **114.8456** |
| 12 | **Pasteuria (treated)** | **Band 7** | 604.7 | 0.918997 | ? | 1.8621386 | **72.80120** |

*Length of the resolving gel = 658

**Table 8: Immunodetection using Col 1982 after 6 h collagenase treatment (Western Blot)**

| S.No. | Lane | Band | Y | Rf | M.W (kDa) | log MW | MW of unknown |
| --- | --- | --- | --- | --- | --- | --- | --- |
| 1 | Molecular marker | Band 1 | 356.9 | 0.543227 | 250 | 2.39794 | -- |
| 2 | Molecular marker | Band 2 | 428.3 | 0.651903 | 150 | 2.176091 | -- |
| 3 | Molecular marker | Band 3 | 529.1 | 0.805327 | 100 | 2 | -- |
| 4 | Molecular marker | Band 4 | 610 | 0.928463 | 75 | 1.875061 | -- |
| 5 | **Pasteuria (untreated)** | **Band 1** | 448.3 | 0.682344 | ? | 2.178281 | **150.7583** |
| 6 | **Pasteuria (treated)** | **Band 2** | 494.5 | 0.752664 | ? | 2.085199 | **121.6744** |
| 7 | **Pasteuria (treated)** | **Band 3** | 587.9 | 0.894825 | ? | 1.89702 | **78.88968** |

*Length of the resolving gel = 657
